# Could calisthenic exercises improve maximal exercise capacity, peripheral muscle strength and quality of life in dyslipidemia?

**DOI:** 10.1371/journal.pone.0326026

**Published:** 2025-06-17

**Authors:** Furkan Özdemir, Melda Sağlam, Aydan Aslı Aksel Uylar, Oğuz A. Uyaroğlu, Nursel Çalik Başaran, Mine Durusu Tanriöver, Naciye Vardar Yağli

**Affiliations:** 1 Department of Physiotherapy and Rehabilitation, Faculty of Health Sciences, Çankırı Karatekin University, Çankırı, Türkiye; 2 Department of Cardiopulmonary Physiotherapy and Rehabilitation, Faculty of Physical Therapy and Rehabilitation, Hacettepe University, Ankara, Türkiye; 3 Department of Internal Disease, Faculty of Medicine, Hacettepe University, Ankara, Türkiye; Ordu University, TÜRKIYE

## Abstract

**Background:**

Regular exercise improves cardiovascular health through regulating the plasma lipoprotein profile. But the effects of different exercise modalities on exercise capacity, muscle strength and quality-of-life in dyslipidemia is unclear.

**Objectives:**

This study aimed to investigate the effects of calisthenic exercises on exercise capacity, muscle strength and quality-of-life in dyslipidemia.

**Methods:**

Thirty-nine individuals were randomly divided into 3 groups: Aerobic + Calisthenic Exercise Group (ACG), Aerobic Exercise Group (AG) and Control Group (CG). Exercise capacity measured using cardiopulmonary exercise test, muscle strength using handheld dynamometer, quality-of-life using Short Form-36 Quality of Life Questionnaire (SF-36) at baseline and 8 weeks after.

**Results:**

Peak oxygen uptake (VO_2peak_) (p < 0.001), metabolic equivalent (MET) (p < 0.001) and percentage of oxygen pulse increased significantly within ACG (p < 0.001). Peak heart rate (p = 0.006) and heart rate reserve improved significantly within AG (p = 0.004). Peak VO_2_ (p < 0.001), MET (p < 0.001), oxygen pulse (p = 0.006), knee extensor (p < 0.001) and handgrip strength decreased significantly within CG (p = 0.009). MET (p < 0.001) and %handgrip strength increased significantly in ACG compared to AG and CG (p = 0.021). Peak VO_2_ (p < 0.001), heart rate (p < 0.001), heart rate reserve (p < 0.001), and handgrip strength increased significantly in ACG compared to CG (p = 0.009). Peak heart rate (p < 0.001), heart rate reserve (p < 0.001) and shoulder abductor strength improved significantly in AG compared to CG (p = 0.019).

**Conclusions:**

Aerobic exercises recommended for regulating blood biochemistry in dyslipidemia. But effects of calisthenic exercises in dyslipidemia are unclear. Our study showed that calisthenic exercises which combined with aerobic exercise training may improve cardiac and pulmonary response to exercise and handgrip strength in dyslipidemia. Additionally, aerobic exercise training may improve cardiac response to exercise in dyslipidemia. **ClinicalTrials Number:** NCT06008912.

## Introduction

Alterations in serum lipid levels are considered a primary or most important factor in various cardiac and metabolic diseases, such as atherosclerosis. Lipid profile disorders, especially elevated total blood cholesterol levels, are a significant public health concern worldwide. Approximately one in every three people is exposed to risk factors that can lead to dyslipidemia [1]. Today, it is reported that ischemic heart and central nervous system diseases are the most important causes of mortality and morbidity globally in the adult population [2]. Lipid profile disorders are recognized as significant risk factors for ischemic heart diseases [1]. These disorders may significantly impact not only the cardiovascular system but also metabolic functions, which lead to various chronic diseases. Cardiovascular structural changes which are associated with hypertension, obesity, or diabetes may affect exercise capacity and these are among the most common comorbidities in dyslipidemia. Dysfunction in vascular endothelial functions and nitric oxide mechanism causes insufficient blood flow in peripheral muscles. As a result, individuals with dyslipidemia may experience decreased exercise capacity, impaired tissue oxygenation and decreased muscle strength, and a diminished quality of life, both due to the condition itself and its associated medical treatments [3,4]. Exercise capacity is an important measure of an individual’s physical fitness. Maximal exercise capacity identifies the peak rate of oxygen consumption during maximal exercise, which describes as peak or maximal oxygen uptake (VO_2peak_). Reduction in exercise capacity is one of the first signs of cardiovascular diseases [5,6].

Since lipid profile disorders are influenced by many different genetic and environmental factors, their incidence varies according to regions, lifestyle habits and individual factors. Apart from individual factors, especially physical activity levels, dietary lipid intake, and deficiencies in other nutrients that balance lipid metabolism, existing comorbid diseases and medical treatments can also change lipid metabolism and may lead to deterioration in the lipid profile [7]. In particular, lifestyle habits appear to be the most modifiable factors affecting lipid metabolism [7]. A multifaceted approach is generally recommended for treating lipid profile disorders. Exercise therapy and dietary counseling are commonly included in these approaches. Exercise therapy is considered a very important treatment option, especially for managing and treating obesity, hypertension, hyperglycemia and metabolic syndrome symptoms that may accompany dyslipidemia [7–9].

Exercise, a structured physical activity targeting specific muscle groups, offers numerous health benefits, including positive impacts on psychological function, quality of life, morbidity, and cardiorespiratory fitness. Physical activity and exercise can boost self-esteem and social participation while reducing depression and other related mental symptoms [10–12]. Calisthenics, a type of exercise that uses body weight as resistance, employs various biomechanical strategies without requiring external equipment. The load effect created by individuals’ body structures and extremities in different positions, and exercises create resistance during movement [13]. The most evident benefit of exercise training, which aerobic or resistance training, is improving exercise tolerance. The most evident results of resistance training in patients with dyslipidemia are the increase in high-density lipoprotein (HDL-C) and decreasing in low-density lipoprotein (LDL-C), triglycerides (TG) and total cholesterol (TK). Additionally, it has stated that resistance training may reduce the risk of cardiovascular disease risk caused by other risk factors such as obesity, diabetes or hypertension in patients with dyslipidemia [14,15]. Effects of calisthenic exercises have explored in different populations such as older adults, athletes, adolescents or healthy adults and different diseases such as COPD or Parkinson’s disease. But the effects of calisthenic exercises, especially in combination with aerobic exercise training, in patients with dyslipidemia are not explored previously. However, there is no data on the effects of calisthenics on exercise capacity, muscle strength, aerobic endurance, physical activity, or quality of life in individuals with dyslipidemia. Thus, the effectiveness of calisthenics as a treatment modality for individuals with dyslipidemia remains unclear.

The study aimed to evaluate the effects of a combined aerobic and calisthenic exercise training program on maximal oxygen consumption, peripheral muscle strength, blood lipids and quality of life in individuals with dyslipidemia. The main target of this study is observing the effects of combining exercise training regimen in mentioned parameters in patients with dyslipidemia.

## Materials and methods

### Study design

A randomized controlled study was conducted at Hacettepe University, Faculty of Physical Therapy and Rehabilitation, Department of Cardiopulmonary Rehabilitation in collaboration with Hacettepe University, Faculty of Medicine, Department of Internal Medicine during the period of 14^th^ May 2023–31th July 2024. The study was approved by the Hacettepe University Clinical Research Ethics Committee with “KA-22027” trial number, and registered to Clinical Trials platform with “NCT06008912” trial number. Written informed consent was obtained from all participants.

### Individuals

Thirty-three participants were included in this study. The inclusion criteria were; age 18–65 years, diagnosed as lipid profile disorders, and being volunteered for participation. The exclusion criteria included recent respiratory infections (within one month), taking blood lipid controlling therapy, a history of malignancy, orthopedic, rheumatological, neurological, cardiac or pulmonary diseases that may affect functional capacity, and any psychiatric or cognitive disorder that could impair cooperation.

The study was planned with a randomized controlled parallel group design. Participants with similar age, gender, and body mass index (BMI) were randomly assigned to one of three groups using a computer-based system. These groups were: an aerobic exercise training group (AG), which exercised three days a week under the supervision of a physiotherapist during eight weeks; an aerobic and calisthenic exercise training group (ACG), which exercised three days a week aerobically and seven days a week with calisthenics, both under physiotherapist supervision during eight weeks; and a control group (CG), which received education on physical activity and personalized physical activity recommendations but no structured exercise program.

### Measurements

#### Maximal exercise capacity.

Maximal exercise capacity was evaluated using cardiopulmonary exercise test (CPET). The test was performed using an analyzer interface (Quark CPET, COSMED®, Rome, Italy) on treadmill. Participants’ electrocardiographic assessment was evaluated using 12 derived electrocardiography system using an analyzer interface. The test was applied according to ATS/ACCP guidelines [16]. VO_2peak_, oxygen pulse (O_2_HR), respiratory exchange ratio (RER), heart rate (HR), heart rate reserve (HRR) and metabolic equivalent (MET) were evaluated periodically every 30 second at resting phase, during the test, at maximum and recovery phase. Additionally, the percentage of predicted VO_2peak_, HR and O_2_HR were evaluated at peak exercise. A physiotherapist continuously monitored the data online through radio transmission. For quality control, gas analyzers and turbine were calibrated before each test. Modified Bruce protocol was applied. Before starting the test, the resting data were recorded for three minutes while the individual was at rest, then the workload was increased every three minutes. The test continued until the individual reached at least 95% of maximum heart rate or RER value reached over 1.1 or individual’s perceived exertion reached over maximal tolerance state. The test was stopped in case any symptoms (shortness of breath, chest pain, fatigue) occurred [16].

#### Peripheral muscle strength.

Participants’ shoulder abductor and knee extensors muscles strength were measured using handheld dynamometers. Participants were asked to maintain maximal isometric contraction against external resistance, and assessments performed according to Bohannon’s “break test” procedure. Handgrip strength (HGS) was assessed with handheld dynamometer, according to Mathiowetz’s assessment procedure. Three assessments were conducted, and the best value was recorded. Reference values were used for comparisons [17,18].

#### Quality of life.

Quality of life was assessed using Turkish version of Short Form 36 Quality of Life Questionnaire (SF-36). SF-36 consists of 36 items and evaluates quality of life in 8 subdomains (physical function, role limitation due to physical problems, pain, general health, vitality/energy, social function, role limitation due to emotional problems, and mental health). Higher score indicates better quality of life [19,20].

#### Serum biochemical analysis.

Serum biochemistry was assessed when diagnosing stage and control appointment by internal medicine physician, after 8 hours of fasting, and then the results were recorded. The serum biochemical analysis consisted of high-density lipoprotein (HDL-C), low-density lipoprotein (LDL-C), total cholesterol (TC), triglycerides (TG), non-HDL cholesterol (Non-HDL-C), C-reactive protein (CRP), hemoglobin-A1C (HbA1C), fasting blood glucose.

### Intervention

Clinical assessment and serum lipid profile analyzes of individuals at the beginning of the study were carried out by internal medicine physician. İndividuals who had been diagnosed as dyslipidemia and are medically fit for exercise training included in the study. İndividuals in ACG received aerobic exercise training as walking in treadmill, with workload of which reaching 60–80% of maximal heart rate, for 40 minutes, three days per a week; and calisthenic exercise training which consists of 9 different exercises (which are triceps dips, push-up, plank, squat, lunge, pelvic bridge, side plank, sit-up, reverse sit-up), with 10 repetitions and 3 session per day, every day during 8 weeks. Aerobic exercise workload was re-determined every week according to heart rate, and calisthenics get advanced according to individual’s perceived exertion. İndividuals in AG received aerobic exercise training as walking on treadmill, with workload of which reaching 60–80% of maximal heart rate, for 40 minutes, three days per a week for 8 weeks. İndividuals in CG received personalized physical activity recommendations and followed up for 8 weeks. All assessments were made for all three groups at the beginning of the study and at the end of the 8th week (after the exercise training) of the study.

### Statistical Analysis

The data obtained in the study were analyzed using SPSS (Statistical Package for Social Sciences) for Windows 22.0 program. In this study, it was calculated that a total of 24 dyslipidemia patients should be included in the study, 8 patients in each group, according to the maximal oxygen consumption value in dyslipidemia patients, with 90% power, 0.82 effect size and 5% type 1 error according to reference study in 2009 whose Shaw et al. [21]. The conformity of variables to normal distribution was examined using visual (histogram and probability graphs) and analytical methods using “Kolmogorov-Smirnov/Shapiro-Wilk tests”. The data were analyzed with IBM SPSS v23. Generalized Estimating Equations (GEE) were used to compare quantitative variables according to Group, Time and Group*Time interactions. Descriptive statistics of quantitative variables were given as mean±standard deviation. Significance level was determined as p < 0.05.

## Results

**Fig 1:**
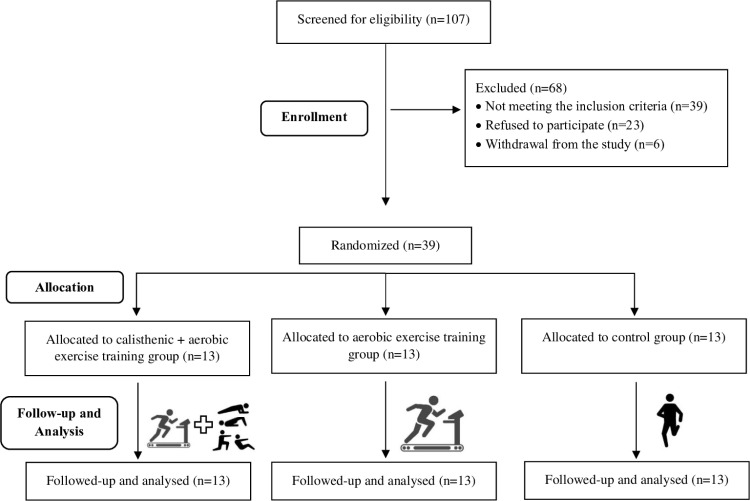
Flow diagram of the participants’ recruitment in the study.

A hundred and seven individuals diagnosed with dyslipidemia were screened. Thirty-nine individuals were excluded because of not meeting inclusion criteria, 23 individuals excluded because of rejecting to participate, and 6 individuals excluded because of withdrawing from study after baseline assessments (Fig 1). Demographic characteristics are similar in all groups and are shown in Table 1. Gender distribution is similar between all groups (p > 0.05). Approximately 77% of the participants in the ACG, approximately 85% of the participants in the AG, and approximately 70% of the participants in the CG were female. The groups were evaluated as overweight and obese in terms of mean BMI [22].

**Table 1:** Demographic characteristics of groups.

		ACG	AG	CG	p
		n(%)	n(%)	n(%)	
**Gender**	**Female**	10(76.9%)	11(84.6%)	9(69.2%)	p = 0.648
	**Male**	3(23.1%)	2(15.4%)	4(30.8%)	
		**Mean±SD**	**Mean±SD**	**Mean±SD**	**p**
**Age (years)**		44.769 ± 9.488	50.231 ± 6.483	46.539 ± 12.421	0.358
**BMI (kg/m**^**2**^)		25.859 ± 7.086	30.618 ± 5.441	25.942 ± 4.148	0.063
Smoking exposure (pack*years)		1.385 ± 4.114	4.539 ± 11.230	6.308 ± 15.697	0.542

n: Sample count, %: Percent, kg: Kilogram, m: Meter, Mean±SD: Mean ± Standart Deviation, ACG: Aerobic + Calisthenic Exercise Group, AG: Aerobic Exercise Group, CG: Control Group, X^2^: Chi-Square Test; One-way ANOVA.

Changes in CPET parameters within and between groups according to before and after intervention shown in Table 2. Total mean RER value for ACG was 1.11, for AG was 1.03 and for CG was 1.16 according to group effect. There was a significant difference between AG and both ACG and CG according to group effect (p < 0.001); but no significant difference between ACG and CG. Total mean RER value for before intervention was 1.1 and after intervention was 1.11 and there was no significant difference according to time effect (p > 0.05). There was no significant difference between groups and time variables according to group*time interaction (p > 0.05). Total mean VO_2_ value for ACG was 24.49, for AG was 20.43 and for CG was 25.33 according to group effect. There was a significant difference between AG and both ACG and CG according to group effect (p < 0.001); but no significant difference between ACG and CG. Total mean VO_2_ value for before intervention was 22.97 and after intervention was 23.86 and there was no significant difference according to time effect (p > 0.05). There was significant difference between groups and time variables according to group*time interaction (p < 0.001). There was a significant difference between AG and CG, but there was no difference between ACG and other groups at before intervention. There was a significant difference between ACG and AG, but no difference between CG and other groups at after intervention in VO_2_. There was a significant difference between before and after intervention in VO_2_ in ACG, but there was no significant difference between before and after intervention in VO_2_ in AG and CG. Total mean %VO_2_ value for ACG was 82.38, for AG was 84.65 and for CG was 79.58 according to group effect. There was no significant difference between groups according to both group effect and time effect (p > 0.05). There was significant difference according to group*time interaction in %VO_2_ (p = 0.007). There was no significant difference in each groups at both before intervention and after intervention in %VO_2_. Total mean MET value for ACG was 6.99, for AG was 5.73 and for CG was 7.25 according to group effect. There was a significant difference between AG and both ACG and CG according to group effect (p < 0.001); but no significant difference between ACG and CG. Total mean MET value for before intervention was 6.58 and after intervention was 6.74 and there was no significant difference according to time effect (p > 0.05). There was significant difference between groups and time variables according to group*time interaction in MET (p < 0.001). There was a significant difference between before and after intervention in ACG. There was a significant difference in both before and after intervention between AG and CG. Total mean HR value for ACG was 159.35, for AG was 162.88 and for CG was 160.85 according to group effect. There was no significant difference between groups according to group effect (p > 0.05). Total mean HR value for before intervention was 159.10 and after intervention was 162.95 and there was a significant difference according to time effect (p = 0.006). There was significant difference between groups and time variables according to group*time interaction in HR (p < 0.001). There was a significant difference between before and after intervention in AG. Total mean %HR value for ACG was 91.27, for AG was 95.58 and for CG was 92.19 according to group effect. There was a significant difference between AG and both ACG and CG according to group effect (p < 0.001); but no significant difference between ACG and CG. Total mean %HR value for before intervention was 91.79 and after intervention was 94.23 and there was a significant difference according to time effect (p = 0.003). There was significant difference between groups and time variables according to group*time interaction in %HR (p < 0.001). There was a significant difference between before and after intervention in AG. Total mean HRR value for ACG was 15.23, for AG was 7.69 and for CG was 13.62 according to group effect. There was a significant difference between AG and both ACG and CG according to group effect (p = 0.001); but no significant difference between ACG and CG. Total mean HRR value for before intervention was 14.15 and after intervention was 10.21 and there was a significant difference according to time effect (p = 0.004). There was significant difference between groups and time variables according to group*time interaction in HRR (p < 0.001). There was a significant difference between before and after intervention in AG. Total mean O_2_HR value for ACG was 10.22, for AG was 9.96 and for CG was 10.02 according to group effect. There was no significant difference between groups according to group effect (p > 0.05). Total mean O_2_HR value for before intervention was 10.09 and after intervention was 10.04 and there was no significant difference according to time effect (p > 0.05). There was significant difference according to group*time interaction in O_2_HR (p = 0.006). There was no significant difference in each groups at both before intervention and after intervention in O_2_HR. Total mean %O_2_HR value for ACG was 91, for AG was 89.6 and for CG was 86.65 according to group effect. There was no significant difference between groups according to group effect (p > 0.05). Total mean %O_2_HR value for before intervention was 89.61 and after intervention was 86.65 and there was no significant difference according to time effect (p > 0.05). There was significant difference according to group*time interaction in O_2_HR (p < 0.001). There was a significant difference in ACG between before and after intervention in O_2_HR.

**Table 2:** Comparisons of the CPET parameters according to group and time effect.

	Aerobic + Calisthenic Exercise Group (n = 13)		Aerobic Exercise Group (n = 13)		Control Group (n = 13)		Group Effect	Time Effect	Group^*^Time Effect
	Baseline	After Training	Baseline	After Training	Baseline	After Training	p	p	p
**RER**	1.12 ± 0.09	1.11 ± 0.11^a^	1.01 ± 0.08	1.05 ± 0.06^a^^,^^c^	1.16 ± 0.14	1.17 ± 0.11^c^	**<0.001** ^*^	0.549	0.282
**VO**_**2peak**_ **(ml/min/kg)**	22.95 ± 4.16^#^	26.04 ± 5.28^a^^,^^#^	19.92 ± 1.75	20.93 ± 2.76^a^^,^^c^	26.05 ± 3.65	24.62 ± 3.88^c^	**<0.001** ^*^	0.062	**<0.001** ^*^
**%VO**_**2peak**_ **(%)**	78.77 ± 13.66	86.00 ± 16.98	84.08 ± 17.78	85.23 ± 12.66	81.92 ± 18.13	77.23 ± 12.01	0.629	0.531	**0.007** ^*^
**MET**	6.56 ± 1.18^#^	7.42 ± 1.49^a^^,^^#^	5.71 ± 0.50	5.75 ± 1.36^a^^,^^c^	7.46 ± 1.02	7.04 ± 1.09^c^	**<0.001** ^*^	0.315	**<0.001** ^*^
**HR (beat/min)**	156.31 ± 15.97	162.38 ± 12.29	159.31 ± 7.32^#^	166.46 ± 6.13^#^	161.69 ± 11.01	160.00 ± 12.97	0.571	**0.006** ^*^	**<0.001** ^*^
**%HR (%)**	89.23 ± 6.30	93.31 ± 5.20^a^	93.46 ± 3.53^#^	97.69 ± 3.99^a^^,^^c^^,^^#^	92.69 ± 2.25	91.69 ± 2.78^c^	**<0.001** ^*^	**0.003** ^*^	**<0.001** ^*^
**HRR (beat/min)**	18.54 ± 10.32	11.92 ± 9.27^a^	11.15 ± 6.20^#^	4.23 ± 7.07^a^^,^^c^^,^^#^	12.77 ± 4.11	14.46 ± 4.77^c^	**0.001** ^*^	**0.004** ^*^	**<0.001** ^*^
**O**_**2**_ **HR (ml/min/beat)**	9.99 ± 1.45	10.44 ± 1.65	10.05 ± 1.31	9.86 ± 1.57	10.22 ± 2.28	9.82 ± 1.98	0.882	0.761	**0.006** ^*^
**%O**_**2**_ **HR (%)**	88.46 ± 15.55	93.75 ± 17.33	91.83 ± 16.15	87.54 ± 14.12	88.69 ± 19.93	84.62 ± 15.19	0.777	0.531	**<0.001** ^*^

RER: Respiratory Exchange Ratio, VO_2peak_: Peak oxygen consumption, MET: Metabolic equivalent, HR: Heart rate, HRR: Heart rate reserve, O_2_HR: Oxygen pulse, ml: Milliliter, min: Minute, kg: Kilogram, %: Percent, Generalized estimating equations, Mean±Standard Deviation.

* p < 0.05.

^a^: Significant difference between Aerobic + Calisthenic Exercise Group and Aerobic Exercise Group according to group effect.

^b^: Significant difference between Aerobic + Calisthenic Exercise Group and Control Group according to group effect.

^c^: Significant difference between Aerobic Exercise Group and Control Group according to group effect.

# : Significant difference between baseline and after training assessment.

Changes in peripheral muscle strength within and between groups according to before and after intervention shown in Table 3. Total mean knee extensor muscle strength value for ACG was 308.14, for AG was 322.75 and for CG was 303.59 according to group effect. There was no significant difference between groups according to group effect (p > 0.05). Total mean knee extensor muscle strength value for before intervention was 308.88 and after intervention was 314.10 and there was no significant difference according to time effect (p > 0.05). There was no significant difference between groups and time variables according to group*time interaction (p > 0.05). Total mean percent of the knee extensor muscle strength value for ACG was 77.45, for AG was 79.79 and for CG was 79.03 according to group effect. There was no significant difference between groups according to group effect (p > 0.05). Total mean percent of the knee extensor muscle strength value for before intervention was 78.70 and after intervention was 78.81 and there was no significant difference according to time effect (p > 0.05). There was no significant difference between groups and time variables according to group*time interaction (p > 0.05). Total mean shoulder abductor muscle strength value for ACG was 180.5, for AG was 167.57 and for CG was 177.20 according to group effect. There was no significant difference between groups according to group effect (p > 0.05). Total mean shoulder abductor muscle strength value for before intervention was 175.36 and after intervention was 174.82 and there was no significant difference according to time effect (p > 0.05). There was a significant difference between groups and time variables according to group*time interaction (p = 0.019). There was no significant difference in each groups at both before intervention and after intervention. Total mean percent of the shoulder abductor muscle strength value for ACG was 114.62, for AG was 104.59 and for CG was 108.16 according to group effect. There was no significant difference between groups according to group effect (p > 0.05). Total mean percent of the shoulder abductor muscle strength value for before intervention was 108.81 and after intervention was 109.44 and there was no significant difference according to time effect (p > 0.05). There was no significant difference between groups and time variables according to group*time interaction (p > 0.05). There was no significant difference in each groups at both before intervention and after intervention. Total mean handgrip strength value for ACG was 32, for AG was 32.77 and for CG was 30.77 according to group effect. There was no significant difference between groups according to group effect (p > 0.05). Total mean handgrip strength value for before intervention was 31.95 and after intervention was 31.74 and there was no significant difference according to time effect (p > 0.05). There was no significant difference between groups and time variables according to group*time interaction (p = 0.009). There was no significant difference in each groups at both before intervention and after intervention. Total mean percent of the handgrip strength value for ACG was 74.5, for AG was 83.73 and for CG was 70.91 according to group effect. There was a significant difference between AG and CG according to group effect (p = 0.021); but no significant difference between ACG and other groups (p > 0.05). Total mean percent of the handgrip strength value for before intervention was 76.79 and after intervention was 75.97 and there was no significant difference according to time effect (p > 0.05). There was no significant difference between groups and time variables according to group*time interaction (p = 0.003). There was no significant difference in each groups at both before intervention and after intervention.

**Table 3:** Comparisons of the peripheral muscle strength according to group and time effect.

	Aerobic + Calisthenic Exercise Group (n = 13)		Aerobic Exercise Group (n = 13)		Control Group (n = 13)		Group Effect	Time Effect	Group^*^Time Effect
	Baseline	After Training	Baseline	After Training	Baseline	After Training	p	p	p
**Dominant KEMS (N)**	301.89 ± 77.14	314.39 ± 58.97	315.17 ± 57.83	330.33 ± 45.30	309.59 ± 76.94	297.59 ± 72.02	0.631	0.471	0.079
**% Dominant KEMS (%)**	75.21 ± 17.31	79.69 ± 13.19	77.89 ± 15.15	81.68 ± 12.68	83.01 ± 25.69	75.05 ± 25.11	0.888	0.964	0.058
**Dominant SAMS (N)**	178.33 ± 54.10	182.67 ± 51.36	162.32 ± 36.17	172.82 ± 28.73	185.42 ± 36.60	168.98 ± 42.69	0.626	0.898	**0.019** ^*^
**% Dominant SAMS (%)**	113.16 ± 23.09	116.08 ± 19.10	101.10 ± 23.29	108.08 ± 19.25	112.17 ± 35.65	104.16 ± 26.35	0.398	0.845	0.222
**Dominant HGS (KgF)**	31.08 ± 7.78	32.92 ± 7.64	33.08 ± 5.85	32.46 ± 6.40	31.69 ± 9.28	29.85 ± 10.38	0.803	0.667	**0.009** ^*^
**%Dominant HGS (%)**	72.18 ± 10.92	76.83 ± 10.06	84.65 ± 13.38	82.80 ± 13.39^c^	73.54 ± 14.14	68.29 ± 12.93^c^	**0.021** ^*^	0.505	**0.003** ^*^

KEMS: Knee Extensor Muscle Strength, SAMS: Shoulder Abductor Muscle Strength, HGS: Handgrip Strength, N: Newton, KgF: Kilogram Force, %: Percent, Generalized estimating equations, Mean±Standard Deviation.

* p < 0.05. ^a^: Significant difference between Aerobic + Calisthenic Exercise Group and Aerobic Exercise Group according to group effect. ^b^: Significant difference between Aerobic + Calisthenic Exercise Group and Control Group according to group effect. ^c^: Significant difference between Aerobic Exercise Group and Control Group according to group effect. ^#^: Significant difference between baseline and after training assessment.

Changes in SF-36 sub-parameters within and between groups according to before and after intervention shown in Table 4. Total mean Physical Function subscale of SF-36 value for ACG was 90.77, for AG was 83.08 and for CG was 72.69 according to group effect. There was a significant difference between ACG and CG according to group effect (p = 0.009); but no significant difference between AG and other groups (p > 0.05). Total mean Physical Function subscale of SF-36 value for before intervention was 81.54 and after intervention was 82.82 and there was no significant difference according to time effect (p > 0.05). There was no significant difference between groups and time variables according to group*time interaction (p > 0.05). There was no significant difference in each groups at both before intervention and after intervention. Total mean Physical Role Limitation subscale of SF-36 value for ACG was 82.69, for AG was 78.85 and for CG was 64.42 according to group effect. There was no significant difference between groups according to group effect (p > 0.05). Total mean Physical Role Limitation subscale of SF-36 value for before intervention was 76.28 and after intervention was 74.36 and there was no significant difference according to time effect (p > 0.05). There was no significant difference between groups and time variables according to group*time interaction (p > 0.05). There was no significant difference in each groups at both before intervention and after intervention. Total mean Pain subscale of SF-36 value for ACG was 75.38, for AG was 70.48 and for CG was 56.54 according to group effect. There was no significant difference between groups according to group effect (p > 0.05). Total mean Pain subscale of SF-36 value for before intervention was 66.41 and after intervention was 68.53 and there was no significant difference according to time effect (p > 0.05). There was no significant difference between groups and time variables according to group*time interaction (p > 0.05). There was no significant difference in each groups at both before intervention and after intervention. Total mean General Health subscale of SF-36 value for ACG was 63.46, for AG was 50.38 and for CG was 58.46 according to group effect. There was no significant difference between groups according to group effect (p > 0.05). Total mean General Health subscale of SF-36 value for before intervention was 56.15 and after intervention was 58.72 and there was no significant difference according to time effect (p > 0.05). There was no significant difference between groups and time variables according to group*time interaction (p > 0.05). There was no significant difference in each groups at both before intervention and after intervention. Total mean Vitality subscale of SF-36 value for ACG was 61.54, for AG was 56.35 and for CG was 49.23 according to group effect. There was no significant difference between groups according to group effect (p > 0.05). Total mean Vitality subscale of SF-36 value for before intervention was 56.15 and after intervention was 55.26 and there was no significant difference according to time effect (p > 0.05). There was no significant difference between groups and time variables according to group*time interaction (p > 0.05). There was no significant difference in each groups at both before intervention and after intervention. Total mean Social Function subscale of SF-36 value for ACG was 86.06, for AG was 87.02 and for CG was 60.10 according to group effect. There was a significant difference between CG and other groups according to group effect (p < 0.001), but no difference between ACG and AG according to group effect (p > 0.05). Total mean Social Function subscale of SF-36 value for before intervention was 76.28 and after intervention was 79.17 and there was no significant difference according to time effect (p > 0.05). There was no significant difference between groups and time variables according to group*time interaction (p > 0.05). There was no significant difference in each groups at both before intervention and after intervention. Total mean Emotional Role Limitation subscale of SF-36 value for ACG was 74.37, for AG was 64.10 and for CG was 51.30 according to group effect. There was no significant difference between groups according to group effect (p > 0.05). Total mean Emotional Role Limitation subscale of SF-36 value for before intervention was 58.98 and after intervention was 67.53 and there was no significant difference according to time effect (p > 0.05). There was no significant difference between groups and time variables according to group*time interaction (p > 0.05). There was no significant difference in each groups at both before intervention and after intervention. Total mean Mental Health subscale of SF-36 value for ACG was 63.54, for AG was 69.08 and for CG was 62.92 according to group effect. There was no significant difference between groups according to group effect (p > 0.05). Total mean Mental Health subscale of SF-36 value for before intervention was 62.97 and after intervention was 67.38 and there was no significant difference according to time effect (p > 0.05). There was no significant difference between groups and time variables according to group*time interaction (p > 0.05). There was no significant difference in each groups at both before intervention and after intervention.

**Table 4:** Comparisons of the SF-36 subscales according to group and time effect.

	Aerobic + Calisthenic Exercise Group (n = 13)		Aerobic Exercise Group (n = 13)		Control Group (n = 13)		Group Effect	Time Effect	Group^*^Time Effect
	Baseline	After Training	Baseline	After Training	Baseline	After Training	p	p	p
**Physical Funciton (0–100)**	91.92 ± 9.69	89.62 ± 23.05^b^	79.62 ± 17.26	86.54 ± 12.97	73.08 ± 19.21	72.31 ± 16.02^b^	**0.009** ^*^	0.600	0.210
**Physical Role Limitation (0–100)**	84.62 ± 24.02	80.77 ± 30.88	75.00 ± 36.80	82.69 ± 27.74	69.23 ± 48.04	59.62 ± 34.67	0.328	0.682	0.341
**Pain (0–100)**	72.12 ± 23.18	78.65 ± 27.85	70.38 ± 20.38	70.58 ± 17.50	56.73 ± 32.49	56.35 ± 29.82	0.183	0.336	0.501
**General Health (0–100)**	60.77 ± 17.30	66.15 ± 19.91	46.54 ± 23.57	54.23 ± 18.69	61.15 ± 15.70	55.77 ± 16.81	0.118	0.388	0.093
**Vitality (0–100)**	59.23 ± 17.54	63.85 ± 21.03	56.54 ± 20.86	56.15 ± 24.59	52.69 ± 21.08	45.77 ± 11.52	0.092	0.769	0.285
**Social Function (0–100)**	80.77 ± 22.02	91.35 ± 21.28^b^	85.58 ± 15.18	88.46 ± 17.28^c^	62.50 ± 27.95	57.69 ± 18.78^b^^,^^c^	**<0.001** ^*^	0.421	0.266
**Emotional Role Limitation (0–100)**	71.80 ± 32.91	76.93 ± 36.98	53.85 ± 44.18	74.35 ± 41.18	51.29 ± 44.34	51.30 ± 37.56	0.138	0.235	0.502
**Mental Health (0–100)**	61.54 ± 22.12	65.54 ± 16.54	66.77 ± 18.65	71.38 ± 20.39	60.62 ± 17.35	65.23 ± 21.63	0.512	0.225	0.995

Generalized estimating equations, Mean±Standard Deviation.

* p < 0.05.

^a^: Significant difference between Aerobic + Calisthenic Exercise Group and Aerobic Exercise Group according to group effect.

^b^: Significant difference between Aerobic + Calisthenic Exercise Group and Control Group according to group effect.

^c^: Significant difference between Aerobic Exercise Group and Control Group according to group effect. ^#^: Significant difference between baseline and after training assessment.

Changes in blood biochemical analysis within and between groups according to before and after intervention shown in Table 5. Total mean LDL value for ACG was 145.96, for AG was 159 and for CG was 149.19 according to group effect. There was no significant difference between groups according to group effect (p > 0.05). Total mean LDL value for before intervention was 154.94 and after intervention was 147.22 and there was a significant difference according to time effect (p = 0.015). There was no significant difference between groups and time variables according to group*time interaction (p > 0.05). There was no significant difference in each groups at both before intervention and after intervention. Total mean HDL value for ACG was 60.81, for AG was 63.12 and for CG was 59.23 according to group effect. There was no significant difference between groups according to group effect (p > 0.05). Total mean HDL value for before intervention was 62.98 and after intervention was 58.91 and there was a significant difference according to time effect (p = 0.002). There was a significant difference between groups and time variables according to group*time interaction (p = 0.02). There was a significant difference in AG between before and after intervention. Total mean TC value for ACG was 222.35, for AG was 226.92 and for CG was 218.5 according to group effect. There was no significant difference between groups according to group effect (p > 0.05). Total mean TC value for before intervention was 224.59 and after intervention was 220.24 and there was no significant difference according to time effect (p > 0.05). There was no significant difference between groups and time variables according to group*time interaction (p > 0.05). There was no significant difference in each groups at both before intervention and after intervention. Total mean TG value for ACG was 115.46, for AG was 132.67 and for CG was 144.62 according to group effect. There was no significant difference between groups according to group effect (p > 0.05). Total mean TG value for before intervention was 129.21 and after intervention was 132.62 and there was no significant difference according to time effect (p > 0.05). There was no significant difference between groups and time variables according to group*time interaction (p > 0.05). There was no significant difference in each groups at both before intervention and after intervention. Total mean Non-HDL-C value for ACG was 166.13, for AG was 222.21 and for CG was 166.96 according to group effect. There was no significant difference between groups according to group effect (p > 0.05). Total mean Non-HDL-C value for before intervention was 192.82 and after intervention was 164.69 and there was no significant difference according to time effect (p > 0.05). There was no significant difference between groups and time variables according to group*time interaction (p > 0.05). There was no significant difference in each groups at both before intervention and after intervention. Total mean CRP value for ACG was 4.4, for AG was 5.72 and for CG was 1.15 according to group effect. There was a significant difference between CG and other groups according to group effect (p = 0.002), but no difference between ACG and AG (p > 0.05). Total mean CRP value for before intervention was 4.33 and after intervention was 3.86 and there was no significant difference according to time effect (p > 0.05). There was a significant difference between groups and time variables according to group*time interaction (p = 0.033). There was a significant difference between AG and CG according to group*time interaction. Total mean fasting blood glucose value for ACG was 91.12, for AG was 98.16 and for CG was 88.65 according to group effect. There was no significant difference between groups according to group effect (p > 0.05). Total mean fasting blood glucose value for before intervention was 91.03 and after intervention was 94.53 and there was no significant difference according to time effect (p > 0.05). There was no significant difference between groups and time variables according to group*time interaction (p > 0.05). There was no significant difference in each groups at both before intervention and after intervention. Total mean HbA1c value for ACG was 5.76, for AG was 5.98 and for CG was 5.76 according to group effect. There was no significant difference between groups according to group effect (p > 0.05). Total mean HbA1c value for before intervention was 5.85 and after intervention was 5.75 and there was a significant difference according to time effect (p = 0.002). There was no significant difference between groups and time variables according to group*time interaction (p > 0.05). There was no significant difference in each groups at both before intervention and after intervention.

**Table 5:** Changes in serum biochemistry within and between groups.

	Aerobic + Calisthenic Exercise Group (n = 13)		Aerobic Exercise Group (n = 13)		Control Group (n = 13)		Group Effect	Time Effect	Group^*^Time Effect
	Baseline	After Training	Baseline	After Training	Baseline	After Training	p	p	p
**LDL-C (mg/dL)**	151.15 ± 29.10^#^	140.77 ± 30.53^#^	159.28 ± 31.71	158.66 ± 31.97	154.38 ± 19.31	144.00 ± 25.09	0.487	**0.015** ^*^	0.137
**HDL-C (mg/dL)**	60.31 ± 10.16	61.31 ± 12.70	65.55 ± 11.22^#^	60.25 ± 10.88^#^	63.08 ± 12.65	55.38 ± 9.99	0.652	**0.002** ^*^	**0.020** ^*^
**TC (mg/dL)**	230.00 ± 41.72	214.69 ± 42.44	219.15 ± 70.52	236.09 ± 41.76	224.62 ± 28.65	212.38 ± 31.01	0.820	0.632	0.309
**TG (mg/dL)**	118.23 ± 67.33	112.69 ± 64.77	126.54 ± 48.55	139.91 ± 45.46	142.85 ± 68.69	146.38 ± 84.55	0.512	0.525	0.278
**Non-HDL-C (mg/dL)**	175.67 ± 34.98	156.58 ± 33.73	249.76 ± 201.79	180.88 ± 38.86	169.23 ± 27.85	164.69 ± 25.46	0.354	0.148	0.212
**CRP (mg/dL)**	5.26 ± 5.81	3.72 ± 4.65^b^	6.69 ± 5.96	4.89 ± 4.29^c^	0.98 ± 1.20	1.47 ± 1.64^b.c^	**0.002** ^*^	0.061	**0.033** ^*^
**Fasting Blood Glucose (mg/dL)**	92.54 ± 15.51	89.69 ± 14.56	92.08 ± 9.23	103.77 ± 38.14	88.54 ± 6.29	88.80 ± 4.85	0.243	0.374	0.264
**HbA1c (%)**	5.84 ± 0.65^#^	5.69 ± 0.58^#^	6.05 ± 0.78^#^	5.91 ± 0.80^#^	5.66 ± 0.47	5.61 ± 0.63	0.436	**0.002** ^*^	0.550

LDL: Low Density Lipoprotein, HDL: High Density Lipoprotein, TK: Total Cholesterol, TG: Triglycerides, CRP: C-reactive Protein, HbA1c: Hemoglobin A1c, Generalized estimating equations, Mean±Standard Deviation.

* p < 0.05. ^a^: Significant difference between Aerobic + Calisthenic Exercise Group and Aerobic Exercise Group according to group effect. ^b^: Significant difference between Aerobic + Calisthenic Exercise Group and Control Group according to group effect. ^c^: Significant difference between Aerobic Exercise Group and Control Group according to group effect.

# : Significant difference between baseline and after training assessment.

## Discussion

This study investigated the effects of combined aerobic and calisthenic exercise versus aerobic-only exercise training on individuals with dyslipidemia. Our findings reveal distinct benefits for each exercise modality in improving various cardiovascular and metabolic parameters. Specifically, the combination of aerobic and calisthenic exercise significantly improved LDL-C, HbA1c and cardiorespiratory fitness parameters, including VO2peak and METmax during CPET. While peripheral muscle strength and overall quality of life did not change significantly within the combined exercise group, this group did show improvements in the physical function and social function dimensions of quality of life, and significantly reduced serum CRP levels compared to the no-exercise control group. In contrast, aerobic-only exercise training significantly improved HDL-C, HbA1c, and several CPET parameters: HR, %HR, and HRR. No significant changes were observed in any parameters within the no-exercise group.

A key finding of our study is that the combined aerobic and calisthenic exercise regimen led to significantly greater improvements in VO_2peak_ and METmax compared to aerobic-only exercise training. Conversely, aerobic-only exercise training resulted in significantly greater improvements in %HR and HRR compared to the combined exercise group. Additionally, aerobic-only exercise training also led to significant improvements in RER, VO_2__peak_, MET, %HR, HRR, %HGS, the social function dimension of quality of life, and CRP levels when compared to the no-exercise group.

Current guidelines and systematic reviews consistently recommend both aerobic and resistance exercise for individuals with dyslipidemia to manage blood lipid concentrations and reduce cardiovascular disease risk [23–25]. However, the specific impact of different exercise combinations on maximal aerobic capacity and muscle strength gains remains a subject of ongoing investigation.

Maximal oxygen consumption, a ccrucial indicator of maximal exercise capacity, significantly improved in the group performing a combination of aerobic and calisthenic exercises. This contrasts with the control group, which experienced a non-statistically significant decrease in VO_2__peak_. Our findings align with previous research. For instance, a 2005 study by Duscha et al. observed an increase in VO_2__peak_ across various aerobic exercise training intensities in overweight individuals with mild to moderate dyslipidemia, while the control group showed no change [26]. Similarly, a training study demonstrated that while both resistance and aerobic exercise groups experienced increases in VO_2__peak_, a combined exercise group showed the most significant improvement in individuals with metabolic syndrome [27]. The observed decline in our control group suggests that aerobic exercise alone may be sufficient to prevent this decline, and the addition of calisthenics further enhances this positive trend. Supporting our results, a 2015 study on overweight adults with moderate to severe dyslipidemia found that a combined aerobic and resistance exercise group exhibited the most significant improvement in VO_2peak_ [28]. Another study on individuals with Type 2 diabetes similarly reported that only the combined exercise group experienced an improvement in VO_2peak_ compared to the control group [29]. These consistent findings underscore the effectiveness of combined exercise in enhancing cardiorespiratory fitness.

The combined exercise group, whose HRR was above the required 15 beats/minute before training, decreased its HRR (to 11 beats/minute post training), and aerobic only exercise training group showed significant improvement in HRR (to 4 beats/minute post training). Post-hoc analysis showed a significant improvement in aerobic only exercise training group compared to the combined exercise and no exercise groups in HRR. This indicates that both physiological and symptomatic exercise tolerance increased in aerobic only exercise training group, and also the aerobic only exercise training group and combined exercise group approached their maximum HR.

Monitoring blood lipids and blood glucose in individuals with dyslipidemia remains a critical area of exercise research. Church et al. found that HbA1c levels significantly improved only in the combined aerobic and resistance exercise group compared to the control group [29]. However, their subgroup analysis revealed significant improvements in both the aerobic and combined groups for individuals with HbA1c levels of 7 or higher, which alignins with our findings. Studies investigating the effects of combined aerobic and resistance exercise on fasting glucose have yielded mixed results. While some studies, such as Bateman et al., found no significant changes in fasting glucose in overweight adults with metabolic syndrome, others have reported improvements in specific subgroups [27]. Our findings are consistent with these studies, indicating that while fasting glucose may not be significantly altered in all individuals, both combined and aerobic exercises can exert beneficial effects on glucose metabolism. HDL-C is the blood lipid parameter most likely to be positively influenced by physical activity and exercise [14]. A study comparing high-intensity aerobic and low-intensity aerobic/resistance exercise combinations in 50 overweight or obese women found significant reductions in TC, LDL-C, and TG, as well as a significant increase in HDL-C, in both groups [30]. Our study’s similar changes in LDL-C in combined exercise group supports these findings; but changes in TC, TG and HDL-C within and between the ACG and AG groups are not as expected in previous studies. TC and TG decreased, and HDL-C increased in combined exercise group but these changes are not statistically significant. Additionally, decrease in HDL-C in all patients according to time effect may indicate that insufficient exercise volume may decrease HDL-C in patients with dyslipidemia. However, the combined training group’s ability to reverse the decline in HDL-C observed in the AG and CG groups is noteworthy. A 2024 study examined the impact of dietary intervention combined with aerobic/resistance exercise on dyslipidemia in men with abdominal obesity. Both interventions resulted in a 14% reduction in LDL-C. The dietary intervention included consuming low-glycemic index carbohydrates, increasing protein intake, and consuming more fiber [31]. This highlights the importance of nutritional monitoring conjunction with exercise for effective blood lipid regulation.

The impact of calisthenic exercise, a form of resistance training, on muscle strength in individuals with dyslipidemia remains relatively unexplored [32]. While calisthenic exercises, which rely on body weight for resistance, may not always allow for individualized load adjustments to optimize muscle strength gains, a 2018 study demonstrated increased muscle strength in both progressive calisthenic and traditional resistance training groups [32]. This study emphasized the importance of modifying body and hand positions to progressively increase the load in healthy individuals [32]. While our ACG group may not have fully optimized the progression of resistance, it’s important to consider that calisthenic exercises often engage multiple muscle groups simultaneously, unlike traditional resistance training, which typically isolates specific muscles [32].

A 2021 study found that older women with dyslipidemia who participated in Pilates sessions, a form of bodyweight exercise, experienced improvements in lower extremity muscle endurance and 6-minute walk test distance [33]. Although no significant between-group differences were observed in our study, the combined exercise group showed an improvement in HGS compared to the aerobic only exercise training and no exercise training group. Interestingly, aerobic only exercise training group showed a decrease in %HGS but the decrease of %HGS in CG is significantly higher. After adjusting for various confounding factors, HGS was found to be inversely associated with dyslipidemia risk in a large Korean population [34]. This suggests that HGS is a valuable parameter for assessing dyslipidemia risk.

A study providing physical activity counseling to individuals with cardiovascular risk factors, including dyslipidemia, did not observe significant improvements in quality of life as measured by the SF-36, despite increased physical activity levels [35]. On the other hand, deterioration in physical functioning and social functioning dimension of quality of life is significantly higher in no exercise group. Given our stringent inclusion and exclusion criteria, our selected population of isolated individuals with dyslipidemia may not have presented significant baseline issues affecting SF-36 scores. This highlights the need for a dyslipidemia-specific quality-of-life questionnaire to accurately assess the impact of interventions on this population.

### Limitations

Several limitations should be considered when interpreting the findings of this study. Firstly, no dietary interventions were implemented, and participants’ dietary habits were not monitored. Incorporating food diaries could have enhanced the study’s power by ensuring adequate calorie and protein intake for muscle building, which is particularly relevant for calisthenic exercises. Secondly, given the nature of calisthenic exercises, exercise intensity was influenced by individual body weight, and progression was subjective, relying on participants’ perceived exertion. While the achieved power was 98% in the study, a larger sample size would have strengthened the findings; therefore, future studies should include larger participant pool. Finally, the intervention timeframe in this study is determined according to the general recommendations for exercise training, typically 8, 12 or 24 weeks. However, all physiological changes observed in this study may be more clearly manifested with longer training periods. Thus, future studies should consider implementing longer exercise training durations.

## Conclusions

The combination of calisthenic exercises with aerobic exercise significantly increased peak oxygen consumption and metabolic equivalent of task during peak exercise compared to aerobic-only exercise training in individuals with dyslipidemia. However, aerobic exercise alone also increased peak heart rate compared to the no-exercise training. Conversely, neither the combined nor aerobic-only exercise interventions significantly improved peripheral muscle strength in individuals with dyslipidemia. While combined calisthenic and aerobic exercises or aerobic-only exercise interventions have not shown consistently significant results across all blood biochemistry parameters, the combination of calisthenic and aerobic exercises significantly decreased low-density lipoprotein in patients with dyslipidemia. Therefore, calisthenic exercise interventions should be included in individuals’ exercise programs alongside aerobic exercise training to enhance exercise tolerance and potentially improve lipid profiles.

## Supporting information

S1 File CONSORT 2010 checklist of information to include when reporting a randomised trial. (PDF)

S2 File ARAŞTIRMA PROTOKOLÜ. (PDF)

S3 File RESEARCH PROTOCOL. (PDF)

S1 Fig Graphical Abstract. (TIF)

S1 Data PONE-Dataset. (XLSX)

## References

[1] GBD 2015 Risk Factors Collaborators. Global, regional, and national comparative risk assessment of 79 behavioural, environmental and occupational, and metabolic risks or clusters of risks, 1990-2015: a systematic analysis for the Global Burden of Disease Study 2015. Lancet. 2016;388(10053):1659–724. doi: 10.1016/S0140-6736(16)31679-8 27733284 PMC5388856

[2] GBD 2019 Diseases and Injuries Collaborators. Global burden of 369 diseases and injuries in 204 countries and territories, 1990-2019: a systematic analysis for the Global Burden of Disease Study 2019. Lancet. 2020;396(10258):1204–22. doi: 10.1016/S0140-6736(20)30925-9 33069326 PMC7567026

[3] Maxwell AJ, Schauble E, Bernstein D, Cooke JP. Limb blood flow during exercise is dependent on nitric oxide. Circulation. 1998;98(4):369–74. doi: 10.1161/01.cir.98.4.369 9711943

[4] Maxwell AJ, Niebauer J, Lin PS, Tsao PS, Bernstein D, Cooke JP. Hypercholesterolemia impairs exercise capacity in mice. Vasc Med. 2009;14(3):249–57. doi: 10.1177/1358863X08100040 19651675 PMC3140166

[5] Castellani JW, Young AJ, Ducharme MB, Giesbrecht GG, Glickman E, Sallis RE. Prevention of cold injuries during exercise. Med Sci Sports Exerc. 2006;38(11):2012–29. doi: 10.1249/01.mss.0000241641.75101.64 17095937

[6] Wagner PD. Determinants of maximal oxygen consumption. J Muscle Res Cell Motil. 2023;44(2):73–88. doi: 10.1007/s10974-022-09636-y 36434438

[7] Tietge UJF. Hyperlipidemia and cardiovascular disease: inflammation, dyslipidemia, and atherosclerosis. Curr Opin Lipidol. 2014;25(1):94–5. doi: 10.1097/MOL.0000000000000051 24398450

[8] De Sousa SMC Dr, Norman RJ Prof. Metabolic syndrome, diet and exercise. Best Pract Res Clin Obstet Gynaecol. 2016;37:140–51. doi: 10.1016/j.bpobgyn.2016.01.006 26972165

[9] Wang Y, Xu D. Effects of aerobic exercise on lipids and lipoproteins. Lipids Health Dis. 2017;16(1):132. doi: 10.1186/s12944-017-0515-5 28679436 PMC5498979

[10] Strunk RC, Mrazek DA, Fukuhara JT, Masterson J, Ludwick SK, LaBrecque JF. Cardiovascular fitness in children with asthma correlates with psychologic functioning of the child. Pediatrics. 1989;84(3):460–4. doi: 10.1542/peds.84.3.460 2771549

[11] Chandratilleke MG, Carson KV, Picot J, Brinn MP, Esterman AJ, Smith BJ. Physical training for asthma. Cochrane Database Syst Rev. 2012;(5):CD001116. doi: 10.1002/14651858.CD001116.pub3 22592674

[12] Eime RM, Young JA, Harvey JT, Charity MJ, Payne WR. A systematic review of the psychological and social benefits of participation in sport for children and adolescents: informing development of a conceptual model of health through sport. Int J Behav Nutr Phys Act. 2013;10:98. doi: 10.1186/1479-5868-10-98 23945179 PMC3751802

[13] Severy ML. Calisthenics. Halls J Health. 189;37:100–3. PMC9248679 36492703

[14] Mann S, Beedie C, Jimenez A. Differential effects of aerobic exercise, resistance training and combined exercise modalities on cholesterol and the lipid profile: review, synthesis and recommendations. Sports Med. 2014;44(2):211–21. doi: 10.1007/s40279-013-0110-5 24174305 PMC3906547

[15] Nazir A, Heryaman H, Juli C, Ugusman A, Martha JW, Moeliono MA, et al. Resistance training in cardiovascular diseases: a review on its effectiveness in controlling risk factors. Integr Blood Press Control. 2024;17:21–37. doi: 10.2147/IBPC.S449086 38523733 PMC10959113

[16] American Thoracic Society, American College of Chest Physicians. ATS/ACCP Statement on cardiopulmonary exercise testing. Am J Respir Crit Care Med. 2003;167(2):211–77. doi: 10.1164/rccm.167.2.211 12524257

[17] Mathiowetz V, Kashman N, Volland G, Weber K, Dowe M, Rogers S. Grip and pinch strength: normative data for adults. Arch Phys Med Rehabil. 1985;66(2):69–74. 3970660

[18] Bohannon RW. Reference values for extremity muscle strength obtained by hand-held dynamometry from adults aged 20 to 79 years. Arch Phys Med Rehabil. 1997;78(1):26–32. doi: 10.1016/s0003-9993(97)90005-8 9014953

[19] Koçyiğit H, Aydemir Ö, Fişek G, Ölmez N, Memiş A. Kısa form-36 (SF-36)’nın türkçe versiyonunun güvenilirliği ve geçerliliği. İlaç ve Tedavi Dergisi. 1999;12:102–6.

[20] Brazier JE, Harper R, Jones NM, O’Cathain A, Thomas KJ, Usherwood T, et al. Validating the SF-36 health survey questionnaire: new outcome measure for primary care. BMJ. 1992;305(6846):160–4. doi: 10.1136/bmj.305.6846.160 1285753 PMC1883187

[21] Shaw I, Shaw BS, Krasilshchikov O. Comparison of aerobic and combined aerobic and resistance training on low-density lipoprotein cholesterol concentrations in men. Cardiovasc J Afr. 200AD;20:290–5. 19907801 PMC3721720

[22] Physical status: the use and interpretation of anthropometry. Report of a WHO Expert Committee. World Health Organ Tech Rep Ser. 1995;854:1–452. 8594834

[23] Sharma S, Pelliccia A, Gati S. The “Ten Commandments” for the 2020 ESC guidelines on sports cardiology and exercise in patients with cardiovascular disease. Eur Heart J. 2021;42(1):6–7. doi: 10.1093/eurheartj/ehaa735 33180902

[24] Madan K, Sawhney JPS. Exercise and lipids. Indian Heart J. 2024;76 Suppl 1(Suppl 1):S73–4. doi: 10.1016/j.ihj.2023.11.270 38599728 PMC11019314

[25] Kodama S, Tanaka S, Saito K, Shu M, Sone Y, Onitake F, et al. Effect of aerobic exercise training on serum levels of high-density lipoprotein cholesterol: a meta-analysis. Arch Intern Med. 2007;167(10):999–1008. doi: 10.1001/archinte.167.10.999 17533202

[26] Duscha BD, Slentz CA, Johnson JL, Houmard JA, Bensimhon DR, Knetzger KJ, et al. Effects of exercise training amount and intensity on peak oxygen consumption in middle-age men and women at risk for cardiovascular disease. Chest. 2005;128(4):2788–93. doi: 10.1378/chest.128.4.2788 16236956

[27] Bateman LA, Slentz CA, Willis LH, Shields AT, Piner LW, Bales CW, et al. Comparison of aerobic versus resistance exercise training effects on metabolic syndrome (from the Studies of a Targeted Risk Reduction Intervention Through Defined Exercise - STRRIDE-AT/RT). Am J Cardiol. 2011;108(6):838–44. doi: 10.1016/j.amjcard.2011.04.037 21741606 PMC3752599

[28] AbouAssi H, Slentz CA, Mikus CR, Tanner CJ, Bateman LA, Willis LH, et al. The effects of aerobic, resistance, and combination training on insulin sensitivity and secretion in overweight adults from STRRIDE AT/RT: a randomized trial. J Appl Physiol (1985). 2015;118(12):1474–82. doi: 10.1152/japplphysiol.00509.2014 25882384 PMC4469920

[29] Church TS, Blair SN, Cocreham S, Johannsen N, Johnson W, Kramer K, et al. Effects of aerobic and resistance training on hemoglobin A1c levels in patients with type 2 diabetes: a randomized controlled trial. JAMA. 2010;304(20):2253–62. doi: 10.1001/jama.2010.1710 21098771 PMC3174102

[30] Said M, Lamya N, Olfa N, Hamda M. Effects of high-impact aerobics vs. low-impact aerobics and strength training in overweight and obese women. J Sports Med Phys Fitness. 2017;57(3):278–88. doi: 10.23736/S0022-4707.16.05857-X 26609965

[31] Suder A, Makiel K, Targosz A, Kosowski P, Malina RM. Positive effects of aerobic-resistance exercise and an ad libitum high-protein, low-glycemic index diet on irisin, omentin, and dyslipidemia in men with abdominal obesity: a randomized controlled trial. Nutrients. 2024;16(20):3480. doi: 10.3390/nu16203480 39458475 PMC11510197

[32] Kotarsky CJ, Christensen BK, Miller JS, Hackney KJ. Effect of progressive calisthenic push-up training on muscle strength and thickness. J Strength Cond Res. 2018;32(3):651–9. doi: 10.1519/JSC.0000000000002345 29466268

[33] Buttelli ACK, Costa RR, Farinha JB, Fagundes A de O, Vieira AF, Barroso BM, et al. Pilates training improves aerobic capacity, but not lipid or lipoprotein levels in elderly women with dyslipidemia: A controlled trial. J Bodyw Mov Ther. 2021;26:227–32. doi: 10.1016/j.jbmt.2020.10.007 33992249

[34] Kim BM, Yi YH, Kim YJ, Lee SY, Lee JG, Cho YH, et al. Association between relative handgrip strength and dyslipidemia in korean adults: findings of the 2014-2015 Korea National Health and Nutrition Examination Survey. Korean J Fam Med. 2020;41(6):404–11. doi: 10.4082/kjfm.19.0073 32045964 PMC7700830

[35] Missud DC, Parot-Schinkel E, Connan L, Vielle B, Huez J-F. Physical activity prescription for general practice patients with cardiovascular risk factors-the PEPPER randomised controlled trial protocol. BMC Public Health. 2019;19(1):688. doi: 10.1186/s12889-019-7048-y 31159805 PMC6547598

