# Supplementary material for: Could calisthenic exercises improve maximal exercise capacity, peripheral muscle strength and quality of life in dyslipidemia?
Source: PLoS One. 2025 Jun 17;20(6):e0326026. doi: 10.1371/journal.pone.0326026 (PMC12173400; doi:10.1371/journal.pone.0326026)
Supplement: S2 File — (PDF) [file pone.0326026.s002.pdf]

ARAŞTIRMA PROTOKOLÜ

1. Araştırmanın Adı:

Dislipidemi tanılı bireylerde aerobik egzersizle kombine edilen kalistenik egzersiz eğitiminin egzersiz toleransı, fiziksel uygunluk ve plazma lipid profili üzerine etkisinin incelenmesi

2. Araştırmanın Gerekçesi:

Lipid profili bozuklukları (özellikle yüksek total kan kolesterol seviyesi), dünya genelinde önemli bir halk sağlığı problemi olarak kabul edilmekte ve yaklaşık olarak her üç kişiden birinin dislipidemiye yol açabilen risk faktörlerine maruz kaldığı bildirilmektedir (1). Dünya genelinde yapılan prevelans araştırmaları ise lipid profili bozukluklarının % 6,9 ile % 43,6 arasında değiştiğini göstermektedir (2–5).

Günümüzde iskemik kalp ve merkezi sinir sistemi hastalıklarının yetişkin popülasyonda küresel çapta mortalite ve morbiditenin en önemli sebepleri olduğu bildirilmektedir (6). Özellikle iskemik kalp hastalıklarına neden olan risk faktörlerinin başında ise lipid profili bozuklukları olduğu kabul edilmektedir (1).

Lipid profili bozuklukları genetik ve çevresel birçok farklı faktörden kaynaklandığından görülme sıklığı bölgelere, yaşam tarzı alışkanlıklarına ve bireysel etkenler göre değişkenlik göstermektedir. Bireysel faktörlerin haricinde özellikle diyette lipid ağırlığı ve lipid metabolizmasını dengeleyici diğer besinlerin eksikliği, fiziksel aktivite seviyesi ve inaktivite, mevcut diğer komorbid hastalıklar ve medikal tedaviler bireylerde lipid metabolizmasını değiştirmekte ve lipid profilinde bozulmaya yol açabilmektedir. Özellikle yaşam tarzı alışkanlıkları lipid metabolizmasını en kolay etkileyebilen ve modifiye edilebilir faktörler olarak karşımıza çıkmaktadır (7).

Genetik faktörlerin etkisini değerlendirmek amacıyla yapılan çalışmalar Kafkas ırklarında kan lipid düzeyinin genel olarak daha yüksek olduğunu (özellikle total plazma kolesterolü), doğu-güney doğu Asya toplumlarında plazmada yüksek yoğunluklu lipoprotein (HDL) miktarının daha az olduğunu, Hispanik kökenli toplumlarda plazma lipid yoğunluğunun genel olarak daha az olduğunu göstermiştir (2,8,9). Bu çalışmalar aynı zamanda aynı bölgede yaşayan diğer etnik gruplarla karşılaştırıldığında lipid profilindeki bu farklılıkların izole olarak incelenen etnik grupta görüldüğünü, dolayısıyla dislipideminin genetik kökeninin de bulunduğunu ifade etmiştir.

Lipid profili bozukluklarının tedavisi yapılırken çok yönlü yaklaşımların izlenmesi önerilmektedir. Diyet danışmanlığı ve egzersiz tedavisinin de bu yaklaşımlarda yer alması genel olarak tavsiye edilmektedir. Egzersiz tedavisi özellikle dislipidemiye eşlik edebilecek obezite, hipertansiyon,

hiperglisemi ve metabolik sendrom belirtilerinin kontrol altına alınabilmesi ve tedavisi için oldukça önemli bir tedavi seçeneği olarak kabul edilmektedir (7,10,11).

Fiziksel aktivite iskelet kası kontraksiyonu oluşturan ve enerji harcamasını artıran vücut hareketleri olarak tanımlanmaktadır (12). Egzersiz ise belirli bir amaca yönelik yapılandırılmış bir program dahilinde belirli kas gruplarını çalıştırmaya odaklanan özelleştirilmiş bir fiziksel aktivite çeşididir. Fiziksel aktivite ve egzersiz sağlık açısından faydalı olup psikolojik fonksiyon, yaşam kalitesi, morbidite ve kardiyorespiratuar uygunluk üzerine pozitif etkisi bulunmaktadır. Fiziksel aktivite ve egzersiz ayrıca özsaygıyı ve sosyal katılımı artırırken depresyon ve ilişkili diğer mental semptomları azaltır (13–15).

Dislipidemi hastalarına uygulanan aerobik egzersiz programlarının hastaların yaşam kalitesini ve fonksiyonelliğini artırdığı bildirilmekle birlikte kalistenik egzersizlerin etkileri hakkında literatür bilgisi bulunmamaktadır. Bu bilgiler ışığında literatür incelendiğinde dislipidemi tanılı bireylerde aerobik egzersizle kombine edilen kalistenik egzersiz eğitiminin lipid profili, egzersiz kapasitesi, doku oksijenizasyonu ve yaşam kalitesi üzerine etkisine dair bilgilerin kısıtlı olduğu görülmektedir.

Araştırmanın Hipotezleri:

H0: Dislipidemili bireylerde aerobik egzersiz eğitimiyle kombine edilen kalistenik egzersiz eğitiminin fiziksel uygunluk, egzersiz toleransı ve kan lipid profili üzerinde etkisi yoktur.

H1: Dislipidemili bireylerde aerobik egzersiz eğitimiyle kombine edilen kalistenik egzersiz eğitiminin fiziksel uygunluk, egzersiz toleransı ve kan lipid profili üzerinde etkisi vardır.

3. Araştırmanın Gereç ve Yöntemi

3.1. Araştırmanın yeri:

Araştırma Hacettepe Üniversitesi Tıp Fakültesi İç Hastalıkları Anabilim Dalı'nda ve Hacettepe Üniversitesi Fizik Tedavi ve Rehabilitasyon Fakültesi'nde yürütülecektir.

3.2. Araştırmanın zamanı:

30.01.2023 – 30.01.2025 tarihleri arasında veri toplanması ve çalışmanın yazılması planlanmaktadır.

3.3. Araştırmanın evreni, örneklemi, araştırma grubu:

Çalışmanın yürütülmesinde sorumlu araştırmacı olarak Uzm. Dr. Oğuz Abdullah Uyaroğlu, yardımcı araştırmacısı ve proje yürütücüsü olarak Prof. Dr. Naciye Vardar Yağlı, yardımcı araştırmacılar olarak Uzm. Fzt. Furkan Özdemir, Prof. Dr. Mine

Durusu Tanrıöver, Uzm. Dr. Nursel Çalık Başaran ve Uzm. Dr. Yahya Doğan görev almaktadır.

Çalışma Dizaynı: Araştırmaya, Hacettepe Üniversitesi Hastanesi İç Hastalıkları (Dahiliye) Anabilim Dalı'ndan dislipidemi tanısıyla yönlendirilen, 18-65 yaş grubu, testlere ve egzersiz eğitimine uyum sağlayabilecek bireylerden gönüllülük esasına göre onam formunu imzalayanlar dahil edilecektir. Aerobik egzersizle kombine edilmiş kalistenik egzersiz grubu, aerobik egzersiz grubu ve fiziksel aktivite önerilerinin yapıldığı dislipidemi tanılı bireyler arasındaki maksimal oksijen tüketimi (VO_{2max}) için çalışma gücü % 90 ve etki büyüklüğü 0,50 için her grupta en az 8 kişi olacak şekilde toplamda 24 kişi olarak örneklem büyüklüğü hesaplanmıştır.

Çalışmaya dahil edilecek bireylerin kriterleri:

- ✓ Dahiliye kliniği tarafından dislipidemi tanısıyla takip ediliyor olmak
- ✓ 18-65 yaş
- ✓ Araştırmaya katılma için gönüllü olma

Çalışmaya katılacak bireylerin dışlama kriterleri:

- ✓ Herhangi bir kardiyak hastalığı bulunmak
- ✓ Eşlik eden bir psikiyatrik hastalığı bulunmak
- ✓ Son 3 ay içerisinde COVID-19 enfeksiyonu geçirmiş olmak
- ✓ Kooperasyonu etkileyebilecek herhangi bir nörolojik problemi bulunmak
- ✓ Fonksiyonel kapasiteyi etkileyebilecek pulmoner veya ortopedik problemi bulunmak

Çalışmanın başlangıcında rutin takibe gelen dislipidemi hastalarının klinik olarak değerlendirilmesi ve rutin biyokimya analizleri Uzm Dr. Oğuz Abdullah Uyaroğlu tarafından yapılacaktır. Egzersiz eğitimine tıbbi açıdan uygun olanlar çalışmaya dahil edilecektir. Bireyler yaş, cinsiyet ve vücut kitle indeksi (VKİ) parametreleri arasından benzer olanlar arasından bilgisayar tabanlı randomizasyon sistemi aracılığıyla rastgele olarak üç gruba ayrılacaktır. Bu gruplar;

- ✓ Fizyoterapist denetiminde haftada 3 gün aerobik egzersiz eğitiminin yapıldığı bireyler
- ✓ Fizyoterapist denetiminde haftada 3 gün aerobik egzersiz eğitimi ve 7 gün kalistenik egzersiz eğitiminin yapıldığı bireyler

- ✓ Fiziksel aktivite öneminin anlatıldığı ve kişiye uygun fiziksel aktivite önerilerinde bulunulduğu bireyler şeklinde olacaktır.

Her üç gruba değerlendirmeler araştırmanın 0. (egzersiz eğitimine başlamadan önce) ve 8. haftası sonunda (egzersiz eğitimi sonrası) olacak şekilde yapılacaktır.
Gönüllüler araştırmacıları (en az 7 iş günü) önceden bildirmek kaydı ile istedikleri takdirde çalışmadan çekilebilirler. Çalışmadan çekilmek istediğini bildiren gönüllülerin 7 iş günü içerisinde son değerlendirmeleri yapılarak elde edilen bulgular kaydedilecektir.

Eğitim süreci içerisinde düzenli değerlendirmeler ile gönüllünün tıbbi durumunun kötüleşmesi, medikal tedavisinin değişmesi ya da sorumlu hekimin gönüllünün araştırmaya devam etmesinin riskli olduğunu bildirmesi halinde ilgili gönüllüler araştırma dışı bırakılabilir.

Araştırmadan çekilen ya da araştırma ekibi tarafından araştırma dışı bırakılan gönüllülerin yerine toplam gönüllü sayısını sağlayacak sayıda yeni gönüllü dahil edilecektir. Araştırmadan çekilen ya da araştırma ekibi tarafından araştırma dışı bırakılan gönüllülerim standart medikal tedavileri ve takibi devam edecektir.

3.4. Araştırmanın tipi:

Randomize (Yaş, cinsiyet ve VKİ'leri benzer olanlar arasından rastgele üç gruba ayrılacaktır.)

3.5. Araştırma için gerekli insan gücü:

Teşhisin konulması ve egzersiz eğitimine uygun hastaların yönlendirilmesi	Uzm. Dr. Oğuz Abdullah Uyaroğlu<br>Uzm. Dr. Nursel Çalık Başaran<br>Prof. Dr. Mine Durusu Tanrıöver
Verilerin toplanması	Uzm. Dr. Oğuz Abdullah Uyaroğlu<br>Uzm. Dr. Nursel Çalık Başaran<br>Prof. Dr. Mine Durusu Tanrıöver<br>Prof. Dr. Naciye Vardar Yağlı<br>Uzm. Fzt. Furkan Özdemir
Egzersiz eğitiminin uygulanması	Prof. Dr. Naciye Vardar Yağlı

	Uzm. Fzt. Furkan Özdemir<br>Uzm. Dr. Yahya Doğan
Verilerin istatistik programa girilmesi	Uzm. Fzt. Furkan Özdemir
Verilerin yorumlanması ve raporlandırılması	Uzm. Dr. Oğuz Abdullah Uyaroğlu<br>Prof. Dr. Naciye Vardar Yağlı<br>Uzm. Fzt. Furkan Özdemir

3.6. Araştırmada toplanacak veriler:

1. Demografik bilgiler: Gönüllülerin ad-soyad, yaş, vücut ağırlığı, boy, dominant taraf, özgeçmiş, soygeçmiş, sigara hikayesi, kullandığı ilaçlar ve hastalık ile ilgili bilgileri kaydedilecektir.

2.Biyokimya: Hastalardan açlık kan örnekleri 8 haftalık eğitim öncesinde ve sonrasında 12 saat açlığın ardından Hacettepe Üniversitesi Hastanesi İç hastalıkları Anabilim Dalı, Genel Dahiliye Bilim Dalı'nda alınacaktır. Eğitim bitiminden 48 saat sonra sabah saatlerinde yapılacaktır. CRP, LDL-C, HDL-C, Apolipoprotein-a, Total kolesterol (TC), Trigliserid (TG), Açlık kan şekeri, HbA1c değerleri kaydedilecektir.

3.Egzersiz toleransı: Kardiyorespiratuar uygunluk koşu bandı ile kardiyopulmoner egzersiz testi (KPET), Quark CPET (Cosmed®, Roma, İtalya) cihazı ile Hacettepe Üniversitesi Fizik Tedavi ve Rehabilitasyon Fakültesi Kardiyopulmoner Rehabilitasyon Ünitesinde yapılacaktır. Test, dezenfekte edilerek yeniden kullanılabilir özellikte olan, burnu ve ağzı sıkıca kapatan plastik maske aracılığıyla ve her solukta ölçüm yöntemi (breath by breath) kullanılarak yapılacaktır. Pandemi koşulları ve göz önünde bulundurularak ve enfeksiyon bulaşını engellemek amacıyla test için kullanılacak maske testten önce ve sonra uygun dezenfektan maddeler ile dezenfekte edilecek ve daha sonra uygun temizleme yöntemleri ile temizlenmesi sağlanacaktır. Maske ve türbinin temizlenmesi için nötr pH değerine sahip (pH=7) yumuşak deterjan kullanılacaktır. Öncelikle ılık su (22°C - 43°C) dolu bir su banyosuna batırılarak temizlenecek yüzeyin tamamen ıslatılması sağlanacaktır. Daha sonra yumuşak deterjan ile ılık suda (22°C - 43°C) yumuşak temizleme fırçaları, yumuşak temizleme süngerleri ve yumuşak bezler aracılığıyla temizlenecek parçanın (maske, türbin, maske sabitleme bantları) yumuşak bir fırça/sünger/bez aracılığıyla üzerindeki görünür kirlerin ovalanarak yüzeyden uzaklaştırılması sağlanacaktır. Maske ve maske sabitleme bantları akar suyun altında en az üç defa üzerindeki kirler ve deterjan kalıntıları giderilene kadar durulanacaktır. Türbinin durulanması için ise en az üç defa temiz su dolu bir kabın içerisinde çalkalama yöntemi

kullanılacaktır. Türbinin temizlenmesi esnasında her seferinde kullanılan su dökülüp temiz su ile bir sonraki durulamaya geçilecektir. Tek kullanımlık kağıt havlular aracılığı ile bütün temizlenen maddeler kurulanacaktır. Maske ve türbin üzerindeki görünür kirler temizlendikten sonra dezenfeksiyon aşamasına geçilecektir. Türbinin dezenfeksiyon işlemi için son 30 gün içerisinde hazırlanmış %1 sodyum hipoklorit (10000 ppm) çözeltisi kullanılacaktır. Çözeltinin hazırlanmasında 4 ölçek suya 1 ölçek çamasır suyu ilave edilecektir. Türbin 20 dakika süreyle dezenfektan çözeltisi içeren kaba batırılacaktır. Daha sonra türbin su ile dolu bir kap içinde durulanacak ve dezenfektanı gidermek için hafifçe sallanacaktır. Türbini kurutmak için antibakteriyel filtre yoluyla kalibrasyon şırıngasına bağlanıp birkaç kez hava çekme-boşaltma yapılacaktır. Türbin temizlendikten sonra, bir sonraki testten önce her zaman kalibrasyonu yapılacaktır. Türbinin temizlenmesi ve dezenfeksiyonu sırasında örnekleme hattının ıslanmamasına dikkat edilecektir. Yüz maskesinin dezenfeksiyon işlemi için sıcak suyla pastörizasyon tekniği kullanılacaktır. Dezenfeksiyon için yüz maskesi ve maskenin sabitleme bantları 71°C - 76°C arasındaki bir sıcaklıkta ayarlanan sıcak su dolu bir su banyosunda 30 dakika süreyle bekletilecektir. Suyun soğumasını önlemek amacıyla 5 dakikada bir suyun sıcaklığı kontrol edilerek yeniden ideal sıcaklığa ulaşması için sıcak su ilave edilecektir. Otuz dakikalık dezenfeksiyon süreci sonrasında sudan kaynaklanan organizmaların gelişmesini engellemek için maske ve maskenin sabitleme bantları iyice kurutulacaktır. Kurutma için tek kullanımlık kâğıt havlu kullanılacaktır. Temizleme ve dezenfeksiyon aşamasında işlemi uygulayan araştırmacı, bulaşı önlemek için koruyucu ekipmanlar (maske, eldiven) kullanacaktır. Araştırmaya katılması için yönlendirilecek gönüllüde tanı konulmuş bulaşıcı bir enfeksiyon varlığı durumunda (tüberküloz, hemoptizi, oral lezyonlar ve diğer bulaşıcı hastalıklar); gönüllü teste yalnız başına alınacak, gönüllünün o gün test uygulanacak son birey olması sağlanacak, glutaraldehit ve sodyum hipoklorit çözeltileri ile gönüllüye uygulanacak testten önce ve hemen sonra tüm yeniden kullanılabilir ve dezenfekte edilebilir yüzeyler ve parçalar temizlenip dezenfekte edilecek, gönüllüye uygulanan test sırasında test yapılan alanın iyi havalandırılmasına dikkat edilecek, testi uygulayacak araştırmacının kişisel koruyucu ekipmanları kullanımına ve enfeksiyon kontrol önlemlerine azami ölçüde dikkat etmesi sağlanacaktır. Ayrıca enfeksiyon bulaşını engellemek amacıyla test sırasında antibakteriyel ve antiviral özellikteki tek kullanımlık filtreler kullanılacaktır. Modifiye Bruce protokolü uygulanarak gönüllülerin hedef kalp hızına ulaşıncaya kadar egzersiz testi devam ettirilecektir. Test sırasında ortaya nadir de olsa çıkabilecek müdahale gerektiren durumlar için güvenlik önlemleri alınacak; adrenalin, defibrilatör ve oksijen kaynağı bulundurulacak ve gönüllü test sırasında Uzm. Dr. Oğuz

Abdullah UYAROĞLU ve Prof. Dr. Naciye VARDAR YAĞLI gözetiminde olacaktır. Egzersiz testi kesin kontraendikasyonları olanlar teste alınmayacaktır. (Akut MI (2 gün içinde), yüksek riskli unstabil anjina, semptomlara veya hemodinamik bozukluğa neden olan kontrol edilemeyen aritmi, aktif endokardit, semptomatik şiddetli aort stenozu,göreceli akut myokardit veya perikardit, güvenli, düzenli ve yeterli test performansını etkileyen fiziksel özür, hastanın izin vermemesi, dekompanse semptomatik kalp yetmezliği, akut pulmoner emboli veya infarktus, egzersiz performansını etkileyebilen akut kardiak olmayan bozukluklar (böbrek yetmezliği..) İskemik göğüs ağrısı, EKG'de iskemik değişiklikler (>2 mm ST elevasyonu), kompleks ektopi (ventriküler taşikardi), 2-3. derece kalp bloğu, SKB'da >20 mmHg ↓, hipertansiyon, ciddi desatürasyon (SpO_2≤% 80 + şiddetli hipoksemi belirti & bulguları, ani solukluk, koordinasyon kaybı, mental konfüzyon, başdönmesi, bayılma, solunum yetmezliği bulguları (siyanoz, ciddi nefes darlığı) görülürse test bitirilecektir. Hastanın teste devam edemeyeceğini ifade ettiği noktada test sonlandırılacaktır. Kardiyorespiratuar uygunluğun en önemli göstergesi olan VO_{2max} değeri ml/kg/dk cinsinden kaydedilecektir.

4. Fiziksel uygunluk değerlendirmesi:

Fonksiyonel kapasite için 1 dakika otur-kalk testi: Gönüllülerden standart bir sandalyeden 1 dakika boyunca duraklamadan oturup kalkması istenecek ve oturup-kalkma sayısı not edilecektir.

Zamanlı kalk-yürü testi: Gönüllülerden oturdukları sandalyeden kalkıp 3 metrelik bir mesafeyi yürümesi ve geri dönüp tekrar oturması istenecek, bu sırada geçen süre not edilecektir.

Periferik kas kuvveti değerlendirmesi: Gönüllülerden hareket boyunca verilecek dirence karşı maksimum izometrik kuvveti üreterek dirence karşı koyması istenecek ve bu esnada açığa çıkan kuvvet bir dinamometre aracılığı ile değerlendirilecektir. Değerlendirme için quadriceps femoris kası, omuz abdüktör kasları ve el kavrama kuvveti kullanılacaktır. El kavrama kuvveti değerlendirilirken el dinamometresini sıkarak oluşturduğu kuvvet ölçülecektir.

Esneklik değerlendirmesi: Gönüllülerden oturdukları yerde ayak parmak uçlarına doğru uzanması istenecektir. Bu sırada gönüllünün el parmak uçları ile ayak parmak uçları arasında kalan mesafe ölçülecektir. Test, 0-50 cm'lik ölçüm cetveli olan standart bir sehpa ile yapılacaktır. Olgu yere oturtulacak ve çıplak ayak tabanlarının düz bir şekilde test sehpasına dayaması sağlanacaktır.

Periferik kas enduransı değerlendirmesi: Gönüllülerden hedef kasların (quadriceps femoris kası, omuz abdüktör kasları) kas kuvvetinin %30'una karşılık gelen ağırlığa karşı yapabildikleri maksimum tekrarlı aktif hareketi yapması istenecektir.

Yaşam kalitesi değerlendirmesi: Gönüllülere verilecek olan SF-36 yaşam kalitesi ölçeğini doldurmaları istenecektir. Ölçek 36 sorudan oluşmaktadır ve doldurulması yaklaşık 10 dakika sürmektedir.

5. Egzersizler

Aerobik egzersizler haftada üç gün, fizyoterapist gözetiminde, bir egzersiz seansı 30 dk sürecek şekilde sekiz hafta süre ile uygulanacaktır. Aerobik egzersiz eğitimi koşubandı üzerinde maksimal kalp hızının % 40-50'sine karşılık gelen iş yükü ile 5 dakikalık bir ısınma periyodu, maksimal kalp hızının %60-80'ine karşılık gelen iş yükünde 20 dakika yükleme ve maksimal kalp hızının % 40-50'sine karşılık gelen iş yükü ile 5 dakikalık bir soğuma periyodu şeklinde uygulanacaktır. Seans, toplam 30 dakika sürecektir.

Kalistenik egzersiz eğitimi, ilgili grupta haftada 3 gün fizyoterapist gözetiminde, 4 gün ev egzersiz programı olarak sekiz hafta boyunca uygulanacaktır. Egzersizler üst ekstremiteye (push-up, plank, triceps dip), alt ekstremiteye (lunge, squat, glute bridge) ve gövdeye yönelik (side plank, leg lift, mekik) bir program şeklinde, hastanın fonksiyonel durumuna göre karar verilerek başlangıç, orta düzey ve ileri düzey zorlukta olarak planlanacaktır. Hastanın fonksiyonel durumundaki gelişime göre egzersizler daha ileri seviye olanlarla değiştirilecektir.

3.7. Verilerin Toplanması:

Veriler olgu rapor formuna işlendikten sonra SPSS programına girilecektir.

3.8. Araştırmadan Beklenen Fayda ve Riskler:

Araştırmanın gönüllüler için herhangi bir risk ya da yan etki oluşturması beklenmemektedir. Bu araştırma ile dislipidemi tanılı bireylerde aerobik egzersizle kombine edilen kalistenik egzersiz programının egzersiz toleransı, kan lipid profili ve fiziksel uygunluk üzerine etkisini gözlemlemek ve elde edilen veriler ışığında dislipidemi tanılı hastalar için rehabilitasyon programlarına yol gösterici bilgiler edinmek hedeflenmektedir.

3.9. Destekleyici:

Hacettepe Üniversitesi Bilimsel Araştırma Projeleri Koordinasyon Birimi

3.10. Araştırma; araştırma protokolü, İyi Klinik Uygulamalar Kılavuzu ve klinik araştırmalar ile ilgili diğer mevzuat ve protokollere uygun biçimde yapılacaktır.

3.11. Verilerin Analizi:

Verilerin istatistiksel analizinde tanımlayıcı istatistik verilecektir. Çalışma öncesi ve sonrası değerlendirilen parametreler arasında üç grup arasındaki fark parametrik dağılımlarda One-Way ANOVA, non-parametrik dağılımlarda Kruskal Wallis Testi kullanılarak değerlendirilecektir. Yanılma olasılığı $p<0.05$ değeri olarak alınacaktır.

Kaynaklar:

1. Forouzanfar MH, Afshin A, Alexander LT, Biryukov S, Brauer M, Cercy K, et al. Global, regional, and national comparative risk assessment of 79 behavioural, environmental and occupational, and metabolic risks or clusters of risks, 1990–2015: a systematic analysis for the Global Burden of Disease Study 2015. Lancet. 2016;388(10053):1659–724.

2. Rivas-Gomez B, Almeda-Valdés P, Tussié-Luna MT, Aguilar-Salinas CA. Dyslipidemia in Mexico, a Call for Action. Rev Investig Clínica [Internet]. 2018 Oct 4;70(5). Available from: http://www.clinicalandtranslationalinvestigation.com/frame_esp.php?id=185

3. Pan L, Yang Z, Wu Y, Yin R-X, Liao Y, Wang J, et al. The prevalence, awareness, treatment and control of dyslipidemia among adults in China. Atherosclerosis [Internet]. 2016 May;248:2–9. Available from: https://linkinghub.elsevier.com/retrieve/pii/S0021915016300466

4. Zhang M, Deng Q, Wang L, Huang Z, Zhou M, Li Y, et al. Prevalence of dyslipidemia and achievement of low-density lipoprotein cholesterol targets in Chinese adults: A nationally representative survey of 163,641 adults. Int J Cardiol [Internet]. 2018 Jun;260:196–203. Available from: https://linkinghub.elsevier.com/retrieve/pii/S0167527317326062

5. Yang F, Ma Q, Ma B, Jing W, Liu J, Guo M, et al. Dyslipidemia prevalence and trends

among adult mental disorder inpatients in Beijing, 2005–2018: A longitudinal observational study. Asian J Psychiatr [Internet]. 2021 Mar;57:102583. Available from: https://linkinghub.elsevier.com/retrieve/pii/S1876201821000393

6. Vos T, Lim SS, Abbafati C, Abbas KM, Abbasi M, Abbasifard M, et al. Global burden of 369 diseases and injuries in 204 countries and territories, 1990–2019: a systematic analysis for the Global Burden of Disease Study 2019. Lancet [Internet]. 2020 Oct;396(10258):1204–22. Available from: https://linkinghub.elsevier.com/retrieve/pii/S0140673620309259

7. Tietge UJF. Hyperlipidemia and cardiovascular disease. Curr Opin Lipidol [Internet]. 2014 Feb;25(1):94–5. Available from: http://journals.lww.com/00041433-201402000-00016

8. Villarreal-Molina MT, Aguilar-Salinas CA, Rodríguez-Cruz M, Riaño D, Villalobos-Comparan M, Coral-Vazquez R, et al. The ATP-binding cassette transporter A1 R230C variant affects HDL cholesterol levels and BMI in the Mexican population: Association with obesity and obesity-related comorbidities. Diabetes. 2007;56(7).

9. Villarreal-Molina MT, Flores-Dorantes MT, Arellano-Campos O, Villalobos-Comparan M, Rodríguez-Cruz M, Miliar-García A, et al. Association of the ATP-binding cassette transporter A1 R230C variant with early-onset type 2 diabetes in a Mexican population. Diabetes. 2008;57(2).

10. De Sousa SMC, Norman RJ. Metabolic syndrome, diet and exercise. Best Pract Res Clin Obstet Gynaecol [Internet]. 2016 Nov;37:140–51. Available from: https://linkinghub.elsevier.com/retrieve/pii/S1521693416000079

11. Wang Y, Xu D. Effects of aerobic exercise on lipids and lipoproteins. Lipids Health Dis [Internet]. 2017 Dec 5;16(1):132. Available from: http://lipidworld.biomedcentral.com/articles/10.1186/s12944-017-0515-5

12. Miles L. Physical activity and health. Nutr Bull [Internet]. 2007 Dec;32(4):314–63. Available from: http://doi.wiley.com/10.1111/j.1467-3010.2007.00668.x

13. Strunk RC, Mrazek DA, Fukuhara JT, Masterson J, Ludwick SK LJ. Cardiovascular fitness in children with asthma correlates with psychologic functioning of the child.

Pediatrics. 1989;84:460–464.

14. Chandratilleke MG, Carson K V., Picot J, Brinn MP, Esterman AJ, Smith BJ. Physical training for asthma (Review). Cochrane Collab. 2013;(9):1–71.

15. Eime R, Young J, Harvey J, Charity M PW. A systematic review of the psychological and social benefits of participation in sport for children and adolescents: informing development of a conceptual model of health through sport. Int J Behav Nutr Phys Act. 2013;10:98.

Sorumlu araştırmacı:

Uzm. Dr. Oğuz Abdullah UYAROĞLU

Yardımcı Araştırmacılar:

Prof. Dr. Mine DURUSU TANRIÖVER

Prof. Dr. Naciye VARDAR YAĞLI

Uzm. Fzt. Furkan ÖZDEMİR

Uzm. Dr. Nursel ÇALIK BAŞARAN

Uzm. Dr. Yahya Doğan
