# Supplementary material for: Could calisthenic exercises improve maximal exercise capacity, peripheral muscle strength and quality of life in dyslipidemia?
Source: PLoS One. 2025 Jun 17;20(6):e0326026. doi: 10.1371/journal.pone.0326026 (PMC12173400; doi:10.1371/journal.pone.0326026)
Supplement: S3 File — (PDF) [file pone.0326026.s003.pdf]

RESEARCH PROTOCOL

1. Name of the Study:

Investigation of the effects of calisthenic exercise training combined with aerobic exercise on exercise tolerance, physical fitness and plasma lipid profile in individuals diagnosed with dyslipidemia.

2. Rationale of the Research:

Lipid profile disorders (especially high total blood cholesterol levels) are considered an important public health problem worldwide and it is reported that approximately one out of every three people is exposed to risk factors that can lead to dyslipidemia (1). Worldwide prevalence studies show that lipid profile disorders vary between 6.9% and 43.6% (2–5).

Today, ischemic heart and central nervous system diseases are reported to be the most important causes of mortality and morbidity globally in the adult population (6). It is accepted that lipid profile disorders are one of the risk factors that cause ischemic heart diseases (1).

Since lipid profile disorders are caused by many different genetic and environmental factors, their incidence varies according to regions, lifestyle habits and individual factors. Apart from individual factors, deficiency of lipid weight and other nutrients that balance lipid metabolism, physical activity level and inactivity, other comorbid diseases and medical treatments change lipid metabolism in individuals and may lead to deterioration in lipid profile. In particular, lifestyle habits are the factors that can easily affect lipid metabolism and can be modified (7).

Studies conducted to evaluate the effect of genetic factors have shown that blood lipid levels are generally higher in Caucasian races (especially total plasma cholesterol), the amount of high-density lipoprotein (HDL) in plasma is less in east-southeast Asian populations, and plasma lipid density is generally lower in populations of Hispanic origin (2,8,9). These studies also stated that these differences in lipid profile compared to other ethnic groups living in the same region were seen in the ethnic group studied in isolation, so there is also a genetic origin of dyslipidemia.

It is recommended to follow multifaceted approaches while treating lipid profile disorders. It is generally recommended that dietary counseling and exercise therapy be included in these approaches. Exercise therapy is considered to be a very important treatment option for the

control and treatment of obesity, hypertension, hyperglycemia and metabolic syndrome symptoms that may accompany dyslipidemia (7,10,11).

Physical activity is defined as body movements that cause skeletal muscle contraction and increase energy expenditure (12). Exercise, on the other hand, is a specialized type of physical activity that focuses on working specific muscle groups within a structured program for a specific purpose. Physical activity and exercise are beneficial for health and have a positive effect on psychological function, quality of life, morbidity and cardiorespiratory fitness. Physical activity and exercise also increase self-esteem and social engagement, while reducing depression and other associated mental symptoms (13–15).

Although aerobic exercise programs applied to dyslipidemia patients are reported to increase the quality of life and functionality of patients, there is no literature information about the effects of calisthenic exercises. In the light of this information, when the literature is examined, it is seen that there is limited information about the effect of calisthenic exercise training combined with aerobic exercise on lipid profile, exercise capacity, tissue oxygenation and quality of life in individuals diagnosed with dyslipidemia.

Hypotheses of the Research:

H0: Calisthenic exercise training combined with aerobic exercise training has no effect on physical fitness, exercise tolerance and blood lipid profile in individuals with dyslipidemia.

H1: Calisthenic exercise training combined with aerobic exercise training has an effect on physical fitness, exercise tolerance and blood lipid profile in individuals with dyslipidemia.

3. Materials and Methods of the Research

3.1. Location of the study:

The research will be carried out at Hacettepe University Faculty of Medicine, Department of Internal Medicine and Hacettepe University Faculty of Physical Therapy and Rehabilitation.

3.2. Time of the research:

It is planned to collect data and write the study between 30.01.2023 and 30.01.2025.

3.3. Universe, sample, research group of the study:

In the execution of the study, Dr. Oğuz Abdullah Uyaroğlu is the responsible researcher, Prof. Dr. Naciye Vardar Yağlı is the assistant researcher and project coordinator, PT Furkan Özdemir, Prof. Dr. Mine Durusu Tanrıöver, Dr. Nursel Çalık Başaran and Dr. Yahya Doğan are working as assistant researchers.

Study Design: The study will include individuals in the 18-65 age group who are referred with the diagnosis of dyslipidemia from the Department of Internal Medicine (Internal Medicine) of Hacettepe University Hospital, and who can adapt to the tests and exercise training, and who signed the consent form on a voluntary basis. A sample size of 24 people was calculated as a total of 24 people, with a working power of 90% and at least 8 people in each group for an effect size of 0.50 for maximal oxygen consumption (VO2max) between calisthenic exercise group combined with aerobic exercise, aerobic exercise group and individuals diagnosed with dyslipidemia for whom physical activity recommendations were made.

Criteria of individuals to be included in the study:

- ✓ Being followed up by the internal medicine clinic with the diagnosis of dyslipidemia
- ✓ 18-65 years old
- ✓ Volunteering to participate in research

Exclusion criteria of individuals to participate in the study:

- ✓ Having any cardiac disease
- ✓ Having a concomitant psychiatric illness
- ✓ Having had a COVID-19 infection in the last 3 months
- ✓ Have any neurological problems that may affect cooperation
- ✓ Having a pulmonary or orthopedic problem that may affect functional capacity

Clinical evaluation and routine biochemistry analyzes of dyslipidemia patients who were followed up routinely at the beginning of the study were performed by Dr. Oğuz Abdullah Uyaroğlu. Those who are medically fit for exercise training will be included in the study. Individuals will be randomly divided into three groups through a computer-based randomization system among those who are similar among age, gender and body mass index (BMI) parameters. These groups are;

- ✓ Individuals who receive aerobic exercise training 3 days a week under the supervision of a physiotherapist
- ✓ Individuals who receive aerobic exercise training 3 days a week and calisthenic exercise training 3 days a week under the supervision of a physiotherapist
- ✓ It will be in the form of individuals in which the importance of physical activity is explained and appropriate physical activity suggestions are made for the person.

Evaluations for all three groups will be made at the end of the 0th week (before starting the exercise training) and 8th week (after the exercise training) of the research.

Volunteers can withdraw from the study if they wish, provided that they notify the researchers (at least 7 working days) in advance. The final evaluations of the volunteers who report that they want to withdraw from the study will be made within 7 working days and the findings will be recorded.

Volunteers may be excluded from the research if the volunteer's medical condition worsens, medical treatment changes, or if the responsible physician reports that the volunteer's continuation of the research is risky with regular evaluations during the training process.

Volunteers who withdraw from the research or are excluded from the research by the research team will be replaced by a number of new volunteers that will ensure the total number of volunteers. Volunteers who withdraw from the research or are excluded from the research by the research team will continue their standard medical treatment and follow-up.

3.4. Type of research:

Randomized (Those with similar age, gender and BMI will be randomly divided into three groups.)

3.5. Manpower required for research:

Diagnosis and referral of patients suitable for exercise training	Dr. Oğuz Abdullah Uyaroğlu<br>Dr. Nursel Çalık Başaran<br>Prof. Dr. Mine Durusu Tanrıöver

Collection of data	Dr. Oğuz Abdullah Uyaroğlu<br>Dr. Nursel Çalık Başaran<br>Prof. Dr. Mine Durusu Tanrıöver<br>Prof. Dr. Naciye Vardar Yağlı<br>Furkan Özdemir
Implementation of exercise training	Prof. Dr. Naciye Vardar Yağlı<br>Furkan Özdemir<br>Dr. Yahya Doğan
Entering the data into the statistical program	Furkan Özdemir
Interpretation and reporting of data	Dr. Oğuz Abdullah Uyaroğlu<br>Prof. Dr. Naciye Vardar Yağlı<br>Furkan Özdemir

3.6. Data to be collected in the research:

1. Demographic information: Volunteers' name-surname, age, body weight, height, dominant side, resume, family history, smoking history, medications used and information about the disease will be recorded.

2. Biochemistry: Fasting blood samples from the patients will be taken from the Hacettepe University Hospital Department of Internal Medicine, Department of General Internal Medicine after 12 hours of fasting before and after 8 weeks of training. It will be held in the morning 48 hours after the end of the training. CRP, LDL-C, HDL-C, Apolipoprotein-a, Total cholesterol (TC), Triglyceride (TG), fasting blood glucose, HbA1c values will be recorded.

3. Exercise tolerance: Cardiopulmonary exercise test (CPET) with cardiorespiratory fitness treadmill will be performed with Quark CPET (Cosmed®, Rome, Italy) device at Hacettepe University Faculty of Physical Therapy and Rehabilitation, Cardiopulmonary Rehabilitation Unit. The test will be carried out by means of a plastic mask that is disinfected and reusable, tightly covers the nose and mouth, and using the breath by breath method. Considering the pandemic conditions and in order to prevent the transmission of infection, the mask to be used for the test will be disinfected with appropriate disinfectants before and after the test, and then it will be cleaned with appropriate cleaning methods. Mild detergent with a neutral pH value (pH = 7) will be used to clean the mask and turbine. First of all, it will be immersed in a water bath filled with warm water (22°C - 43°C) to ensure that the surface to be cleaned is

completely wetted. Then, the visible dirt on the part to be cleaned (mask, turbine, mask fixing tapes) will be removed from the surface by rubbing the visible dirt on it by means of a soft brush/sponge/cloth in warm water (22°C - 43°C) with mild detergent (22°C - 43°C). The mask and mask fixing tapes will be rinsed at least three times under running water until dirt and detergent residues are removed. For rinsing the turbine, the method of rinsing at least three times in a container filled with clean water will be used. During the cleaning of the turbine, the water used each time will be poured and the next rinse will be started with clean water. All cleaned items will be dried with disposable paper towels. After the visible dirt on the mask and turbine is cleaned, the disinfection phase will be started. For the disinfection process of the turbine, 1% sodium hypochlorite (10000 ppm) solution prepared within the last 30 days will be used. In the preparation of the solution, 1 part bleach will be added to 4 parts water. The turbine will be immersed in the container with disinfectant solution for 20 minutes. The turbine will then be rinsed in a container filled with water and gently shaken to remove the disinfectant. In order to dry the turbine, it will be connected to the calibration syringe through the antibacterial filter and air extraction-discharge will be done several times. Once the turbine has been cleaned, it will always be calibrated before the next test. Care will be taken to ensure that the sampling line does not get wet during cleaning and disinfection of the turbine. Hot water pasteurization technique will be used for the disinfection process of the face mask. For disinfection, the face mask and the fixing tapes of the mask will be kept for 30 minutes in a water bath filled with hot water set at a temperature between 71°C - 76°C. In order to prevent the water from cooling, the temperature of the water will be checked every 5 minutes and hot water will be added to reach the ideal temperature again. After a thirty-minute disinfection process, the mask and the fixing tapes of the mask will be thoroughly dried to prevent the development of waterborne organisms. Disposable paper towels will be used for drying. During the cleaning and disinfection phase, the researcher performing the process will use protective equipment (mask, gloves) to prevent contamination. In the presence of a diagnosed infectious infection in the volunteer to be directed to participate in the study (tuberculosis, hemoptysis, oral lesions and other infectious diseases); The volunteer will be taken to the test alone, it will be ensured that the volunteer is the last individual to be tested that day, all reusable and disinfectable surfaces and parts will be cleaned and disinfected before and immediately after the test to be applied to the volunteer with glutaraldehyde and sodium hypochlorite solutions, attention will be paid to good ventilation of the tested area during the test applied to the volunteer, the use of personal protective equipment by the researcher who will perform the test and infection control It will be ensured that he pays

maximum attention to his precautions. In addition, disposable filters with antibacterial and antiviral properties will be used during the test to prevent infection transmission. By applying the modified Bruce protocol, the exercise test will be continued until the volunteers' target heart rate is reached. Safety precautions will be taken for situations that require intervention that may arise during the test, albeit rarely; adrenaline, defibrillator and oxygen supply will be available and will be under the supervision of Specialist Dr. Oğuz Abdullah UYAROĞLU and Prof. Dr. Naciye VARDAR YAĞLI during the voluntary test. Those who have definite contraindications to the exercise test will not be admitted to the test. (Acute MI (within 2 days), high-risk unstable angina, uncontrollable arrhythmia causing symptoms or hemodynamic disturbance, active endocarditis, symptomatic severe aortic stenosis, relative acute myocarditis or pericarditis, physical disability affecting safe, regular, and adequate test performance, patient disapproval, decompensated symptomatic heart failure, acute pulmonary embolism or infarction, acute non-cardiac disorders that may affect exercise performance (renal failure..) If ischemic chest pain, ischemic changes on ECG (>2 mm ST elevation), complex ectopia (ventricular tachycardia), 2nd-3rd degree heart block, >20 mmHg in SBB↓, hypertension, severe desaturation (SpO_2 ≤80% + severe hypoxemia signs & symptoms, sudden pallor, loss of coordination, mental confusion, dizziness, fainting, signs of respiratory failure (cyanosis, severe shortness of breath) are seen, the test will be completed. The test will be terminated at the point where the patient expresses that he cannot continue the test. The VO 2max value, which is the most important indicator of cardiorespiratory fitness, will be recorded in ml/kg/min.

4. Physical fitness assessment:

1-minute sit-stand test for functional capacity: Volunteers will be asked to sit and stand up from a standard chair for 1 minute without pausing, and the number of sit-stand sessions will be noted.

Timed get-and-walk test: Volunteers will be asked to get up from their chair, walk a distance of 3 meters and return and sit down again, while the time elapsed will be noted.

Peripheral muscle strength evaluation: Volunteers will be asked to resist the resistance by producing the maximum isometric force against the resistance to be given throughout the movement, and the force released during this time will be evaluated by means of a dynamometer. Quadriceps femoris muscle, shoulder abductor muscles and hand grip strength will be used for evaluation. When evaluating the hand grip force, the force created by squeezing the hand dynamometer will be measured.

Flexibility assessment: Volunteers will be asked to lie down on their toes where they sit. At this time, the distance between the fingertips and toes of the volunteer will be measured. The test will be carried out with a standard stand with a 0-50 cm measuring ruler. The patient will be placed on the floor and the soles of the bare feet will be placed flat on the test table.

Peripheral muscle endurance assessment: Volunteers will be asked to perform the maximum repetitive active movement of the target muscles (quadriceps femoris muscle, shoulder abductor muscles) against the weight corresponding to 30% of the muscle strength.

Quality of life assessment: Volunteers will be asked to complete the SF-36 quality of life scale. The scale consists of 36 questions and takes about 10 minutes to fill out.

5. Exercises

Aerobic exercises will be performed three days a week, under the supervision of a physiotherapist, for eight weeks, with an exercise session lasting 30 minutes. Aerobic exercise training will be applied on the treadmill in the form of a 5-minute warm-up period with a workload corresponding to 40-50% of the maximal heart rate, 20 minutes of loading at a workload corresponding to 60-80% of the maximal heart rate, and a 5-minute cool-down period with a workload corresponding to 40-50% of the maximal heart rate. The session will last a total of 30 minutes.

Calisthenic exercise training will be applied for eight weeks in the relevant group 3 days a week under the supervision of a physiotherapist. Exercises will be planned in the form of a program for the upper extremity (push-up, plank, triceps bottom), lower extremity (lunge, squat, glute bridge) and trunk (side plank, leg lift, sit-up), according to the functional status of the patient, and will be planned as beginner, intermediate and advanced difficulty. Depending on the development of the patient's functional status, the exercises will be replaced with more advanced ones.

3.7. Data Collection:

After the data is processed in the case report form, it will be entered into the SPSS program.

3.8. Expected Benefits and Risks from the Research:

The study is not expected to pose any risks or side effects to the volunteers. With this study, it is aimed to observe the effect of calisthenic exercise program combined with aerobic exercise on exercise tolerance, blood lipid profile and physical fitness in

individuals diagnosed with dyslipidemia and to obtain information that guides rehabilitation programs for patients diagnosed with dyslipidemia in the light of the data obtained.

3.9. Supporting:

Hacettepe University Scientific Research Projects Coordination Unit

3.10. Research:

The research protocol will be carried out in accordance with the Good Clinical Practice Guide and other legislation and protocols related to clinical research.

3.11. Analysis of Data:

Descriptive statistics will be given in the statistical analysis of the data. The difference between the three groups between the parameters evaluated before and after the study will be evaluated by using One-Way ANOVA in parametric distributions and Kruskal Wallis Test in non-parametric distributions. The probability of error will be taken as a value of $p<0.05$.

References:

1. Forouzanfar MH, Afshin A, Alexander LT, Biryukov S, Brauer M, Cercy K, et al. Global, regional, and national comparative risk assessment of 79 behavioural, environmental and occupational, and metabolic risks or clusters of risks, 1990–2015: a systematic analysis for the Global Burden of Disease Study 2015. Lancet. 2016; 388(10053):1659–724.
2. Rivas-Gomez B, Almeda-Valdés P, Tussié-Luna MT, Aguilar-Salinas CA. Dyslipidemia in Mexico, a Call for Action. Rev Investig Clinica [Internet]. 2018 Oct 4; 70(5). Available from: http://www.clinicalandtranslationalinvestigation.com/frame_esp.php?id=185
3. Pan L, Yang Z, Wu Y, Yin R-X, Liao Y, Wang J, et al. The prevalence, awareness, treatment and control of dyslipidemia among adults in China. Atherosclerosis [Internet]. 2016 May; 248:2–9. Available from: https://linkinghub.elsevier.com/retrieve/pii/S0021915016300466

4. Zhang M, Deng Q, Wang L, Huang Z, Zhou M, Li Y, et al. Prevalence of dyslipidemia and achievement of low-density lipoprotein cholesterol targets in Chinese adults: A nationally representative survey of 163,641 adults. Int J Cardiol [Internet]. 2018 Jun; 260:196–203. Available from: https://linkinghub.elsevier.com/retrieve/pii/S0167527317326062

5. Yang F, Ma Q, Ma B, Jing W, Liu J, Guo M, et al. Dyslipidemia prevalence and trends among adult mental disorder inpatients in Beijing, 2005–2018: A longitudinal observational study. Asian J Psychiatr [Internet]. 2021 Mar;57:102583. Available from: https://linkinghub.elsevier.com/retrieve/pii/S1876201821000393

6. Vos T, Lim SS, Abbafati C, Abbas KM, Abbasi M, Abbasifard M, et al. Global burden of 369 diseases and injuries in 204 countries and territories, 1990–2019: a systematic analysis for the Global Burden of Disease Study 2019. Lancet [Internet]. 2020 Oct; 396(10258):1204–22. Available from: https://linkinghub.elsevier.com/retrieve/pii/S0140673620309259

7. Tietge UJF. Hyperlipidemia and cardiovascular disease. Curr Opin Lipidol [Internet]. 2014 Feb; 25(1):94–5. Available from: http://journals.lww.com/00041433-201402000-00016

8. Villarreal-Molina MT, Aguilar-Salinas CA, Rodríguez-Cruz M, Riaño D, Villalobos-Comparan M, Coral-Vazquez R, et al. The ATP-binding cassette transporter A1 R230C variant affects HDL cholesterol levels and BMI in the Mexican population: Association with obesity and obesity-related comorbidities. Diabetes. 2007; 56(7).

9. Villarreal-Molina MT, Flores-Dorantes MT, Arellano-Campos O, Villalobos-Comparan M, Rodríguez-Cruz M, Miliar-García A, et al. Association of the ATP-binding cassette transporter A1 R230C variant with early-onset type 2 diabetes in a Mexican population. Diabetes. 2008; 57(2).

10. De Sousa SMC, Norman RJ. Metabolic syndrome, diet and exercise. Best Pract Res Clin Obstet Gynaecol [Internet]. 2016 Nov; 37:140–51. Available from: https://linkinghub.elsevier.com/retrieve/pii/S1521693416000079

11. Wang Y, Xu D. Effects of aerobic exercise on lipids and lipoproteins. Lipids Health Dis [Internet]. 2017 Dec 5; 16(1):132. Available from: http://lipidworld.biomedcentral.com/articles/10.1186/s12944-017-0515-5

12. Miles L. Physical activity and health. Nutr Bull [Internet]. 2007 Dec; 32(4):314–63. Available from: http://doi.wiley.com/10.1111/j.1467-3010.2007.00668.x

13. Strunk RC, Mrazek DA, Fukuhara JT, Masterson J, Ludwick SK LJ. Cardiovascular fitness in children with asthma correlates with psychologic functioning of the child. Pediatrics. 1989; 84:460–464.

14. Chandratilleke MG, Carson K V., Picot J, Brinn MP, Esterman AJ, Smith BJ. Physical training for asthma (Review). Cochrane Collab. 2013; (9):1–71.

15. Eime R, Young J, Harvey J, Charity M PW. A systematic review of the psychological and social benefits of participation in sport for children and adolescents: informing development of a conceptual model of health through sport. Int J Behav Nutr Phys Act. 2013;10:98.

Principal investigator:

Exp. Dr. Oğuz Abdullah UYAROĞLU

Co-Investigators:

Prof. Mine DURUSU TANRIÖVER, MD

Prof. Naciye VARDAR YAĞLI, MD

Exp. Furkan ÖZDEMİR

Exp. Dr. Nursel ÇALIK BAŞARAN

Exp. Dr. Yahya Doğan
