# Supplementary figures and images for: Could calisthenic exercises improve maximal exercise capacity, peripheral muscle strength and quality of life in dyslipidemia?
Source: PLoS One. 2025 Jun 17;20(6):e0326026. doi: 10.1371/journal.pone.0326026 (PMC12173400; doi:10.1371/journal.pone.0326026)

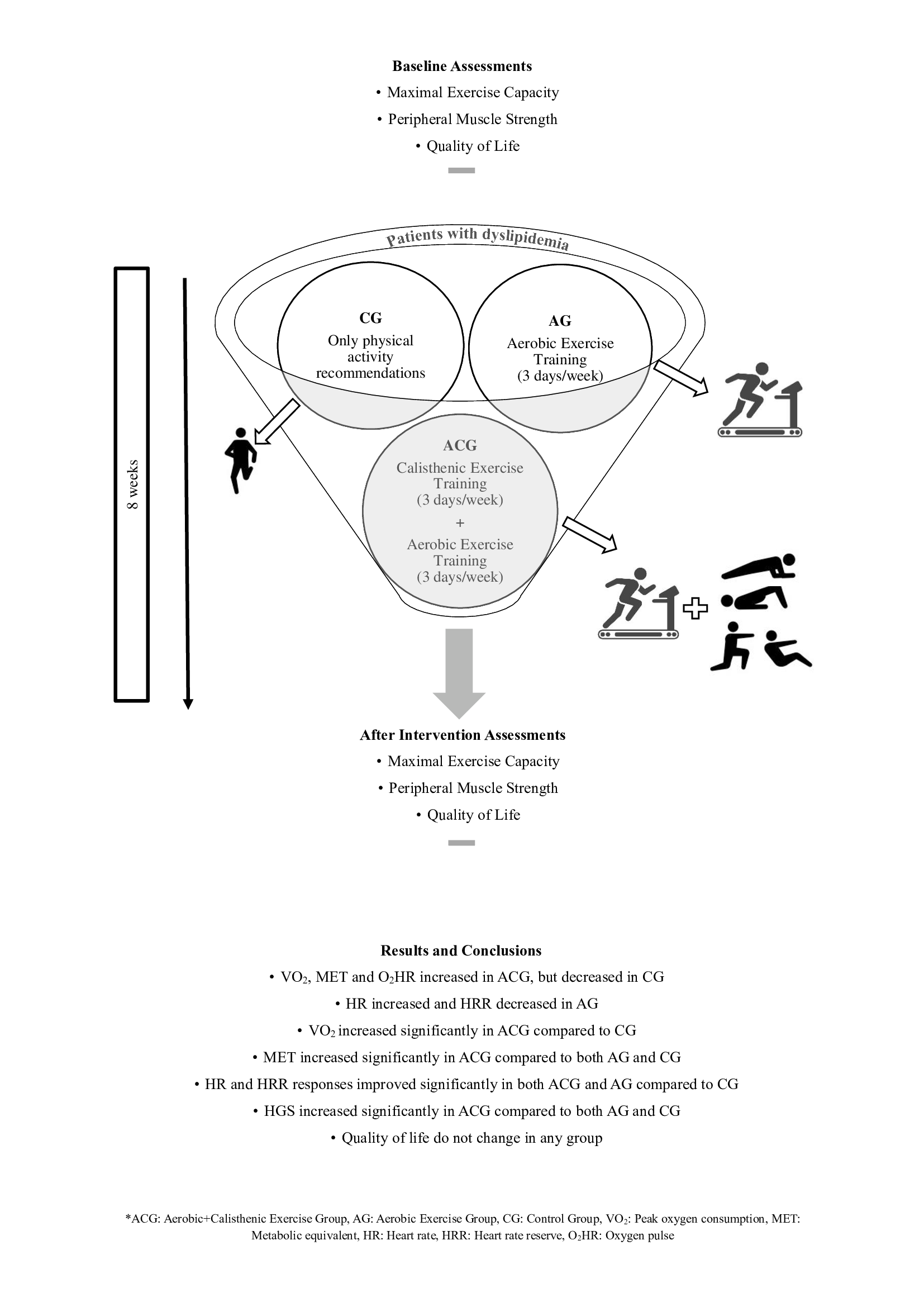

Supplement: S1 Fig — (TIF) [file pone.0326026.s004.tif]
